# Deep Analysis of Residue Constraints (DARC): identifying determinants of protein functional specificity

**DOI:** 10.1038/s41598-019-55118-6

**Published:** 2020-02-03

**Authors:** Farzaneh Tondnevis, Elizabeth E. Dudenhausen, Andrew M. Miller, Robert McKenna, Stephen F. Altschul, Linda B. Bloom, Andrew F. Neuwald

**Affiliations:** 1 Biochemistry and Molecular Biology, University of Florida, PO BOX 100245, Gainesville, Florida 32610 USA; 2 National Center for Biotechnology Information, National Library of Medicine, National Institutes of Health, Building 38A, 8600 Rockville Pike, Bethesda, MD 20894 USA; 3 Institute for Genome Sciences and Department of Biochemistry & Molecular Biology, University of Maryland School of Medicine, 670W. Baltimore Steet, Baltimore, MD 21201 USA

**Keywords:** Cell-cycle proteins, Protein sequence analyses

## Abstract

Protein functional constraints are manifest as superfamily and functional-subgroup conserved residues, and as pairwise correlations. Deep Analysis of Residue Constraints (DARC) aids the visualization of these constraints, characterizes how they correlate with each other and with structure, and estimates statistical significance. This can identify determinants of protein functional specificity, as we illustrate for bacterial DNA clamp loader ATPases. These load ring-shaped sliding clamps onto DNA to keep polymerase attached during replication and contain one δ, three γ, and one δ’ AAA+ subunits semi-circularly arranged in the order δ-γ_1_-γ_2_-γ_3_-δ’. Only γ is active, though both γ and δ’ functionally influence an adjacent γ subunit. DARC identifies, as functionally-congruent features linking allosterically the ATP, DNA, and clamp binding sites: residues distinctive of γ and of γ/δ’ that mutually interact in trans, centered on the catalytic base; several γ/δ’-residues and six γ/δ’-covariant residue pairs within the DNA binding N-termini of helices α2 and α3; and γ/δ’-residues associated with the α2 C-terminus and the clamp-binding loop. Most notable is a trans-acting γ/δ’ hydroxyl group that 99% of other AAA+ proteins lack. Mutation of this hydroxyl to a methyl group impedes clamp binding and opening, DNA binding, and ATP hydrolysis—implying a remarkably clamp-loader-specific function.

## Introduction

An important question in biology is which sequence and structural features enable proteins sharing a common catalytic core to perform entirely different functions. Consider, for example, AAA+ ATPases, which mediate a wide variety of cellular activities, including membrane fusion, DNA replication, microtubule dynamics, intracellular transport, transcriptional activation, protein refolding or degradation, and the disassembly of protein complexes^1,2^. These form homomeric or heteromeric complexes consisting of from five to seven AAA+ modules with ATP-binding sites typically interacting with an adjacent module. Each complex channels the energy of ATP hydrolysis into coordinated conformational changes specific to its function. Although we cannot directly observe the biochemical mechanisms mediating these processes, given enough sequence data we can infer mechanistically imposed constraints. The nature of these constraints varies. They may appear as residues conserved in an entire superfamily or in functionally related protein subgroups (i.e., as correlations between sequence patterns and biochemical properties), as subtle pairwise correlations, or as correlations among these sequence features or with structural features.

Previously investigated protein constraints include function determining residues (FDRs), “coevolving sectors”, directly coupled (DC) residue pairs, and subgroup-specific patterns. FDR methods^3–24^ generally focus on predicting specific, well-characterized residue functions, such as in substrate recognition and catalysis, that can be benchmarked experimentally^25^. However, due to the incompleteness of experimental annotations, we lack reliable gold standards for important but as yet undiscovered residue functions^26,27^, which, along with objectively characterizing protein constraints, is the focus of our investigation here. One may uncover new residue functions by applying statistical methods—which most FDR approaches lack—to distinguish signal from noise within large data sets. Methods that focus on evolutionary changes within a phylogenetic tree^28,29^ make no presuppositions concerning residue functions, but cannot adequately analyze large numbers of sequences due to the need to construct a tree, which, for large data sets, introduces more complexity than either is necessary or can be reliably inferred.

Statistical Coupling Analysis (SCA)^30^ applies principal component analysis to a multiple sequence alignment (MSA) covariance matrix to identify groups of “coevolving protein sectors”^31^ that are believed to arise from selection acting upon protein functional properties^32^. SCA has been used to predict hydrophobic cavities^33^ and surface sites^34^ involved in allosteric regulation and to design proteins^35^. However, most published SCA studies identify a single sector, for which, it has been suggested^36^, statistically equivalent predictions may be made using sequence conservation alone. If so, then SCA may be most useful when multiple sectors are present.

Direct coupling analysis (DCA)^37^ is similar to SCA but uses a different algorithmic approach^38^ and therefore extracts different biologically relevant information^39^. DCA focuses on predicting contacts between residue pairs based on correlated substitution patterns among homologous proteins: In order to maintain structural integrity, substitutions at one residue position often result in compensating substitutions at other positions over evolutionary time. Hence, in principle, MSA covariance analysis can predict structural contacts. However, early approaches fell short of expectations due to the confounding effect of indirect correlations: When residues correlate both at positions *i* and *j* and at positions *j* and *k*, then residues at positions *i* and *k* may also correlate even though they fail to interact directly. DCA^39–46^ overcomes this problem by disentangling direct from indirect correlations. DCA employs a variety of algorithmic strategies, including sparse inverse covariance estimation^41^, multivariate Gaussian modeling^47^, and pseudo-likelihood maximum entropy optimization^43,44,48^; among these, the last strategy (as implemented in CCMpred^48^) performed best based on the estimated significance of the overlap between high DC-scores and 3D-contacts^49^.

Bayesian Partitioning with Pattern Selection (BPPS)^50–52^, like DCA, identifies correlations among columns in an MSA, but unlike DCA, focuses on residues co-conserved among functionally related subgroups. Using Markov chain Monte Carlo sampling, BPPS partitions an MSA into hierarchically arranged subgroups, each defined by a corresponding conserved pattern that best distinguishes that subgroup from those further up the hierarchy. The overlap is typically weak between BPPS pattern residues and either FDRs^53^ or high scoring DCA residue pairs^49^. Moreover, as illustrated here, BPPS enhances the utility of DCA by allowing characterization of direct couplings specific to a functionally-divergent subgroup. Hence, DCA and BPPS are complementary, with a combined analysis often providing deeper biological insight.

Here we describe Deep Analysis of Residue Constraints (DARC), which applies unsupervised machine learning based on both Bayesian and frequentist statistical modeling to perform a multifaceted analysis of residue constraints. It applies regularization methods to avoid over-fitting during model selection. When applied to bacterial DNA clamp loader AAA+ subunits, DARC reveals highly distinctive, biologically interpretable features, the most striking of which is a hydroxyl group that interacts in trans with an adjacent active site. Biochemical analyses reveal that this hydroxyl group is involved in key DNA clamp-loader-specific functions.

## Methods

### Deep analysis of residue constraints

**Figure 1:**
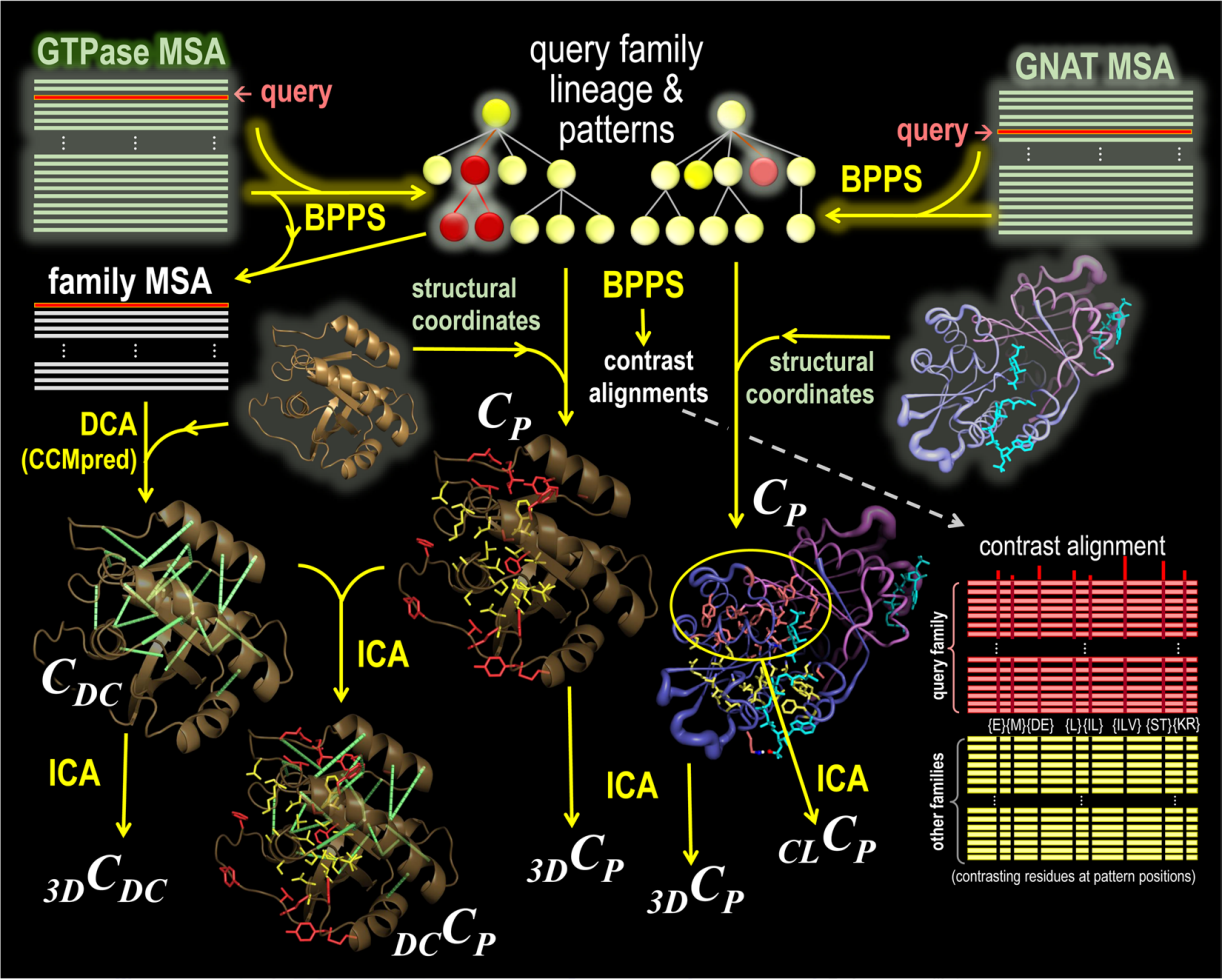
DARC workflow diagram. Algorithmic steps are illustrated schematically using two distinct and independent analyses: on the left, an analysis of P-loop GTPases that shows the steps to estimate *C*_*DC*_, _*3D*_*C*_*DC*_ and _*DC*_*C*_*P*_ with Ras GTPase (pdbid: 1ctq) as the query; and, on the right, an analysis of GCN5-related N-acetyltransferases (GNAT) that shows the steps to estimate _*CL*_*C*_*P*_ with Gna1 (pdbid: 4ag9) as the query; both analyses show the steps to estimate *C*_*P*_ and _*3D*_*C*_*P*_. The input, computational operations, and output are indicated by green, yellow, and white text, respectively. First, DARC applies BPPS to define the query protein lineage (within an implicit superfamily hierarchy) based on discriminating pattern residues, which correspond to *C*_*P*_ constraints and which are visualized both in available 3D structures and in a contrast alignment. Note that, for clarity, each lineage in this diagram only extends to the query family level, even though DARC can extend the lineage to the subfamily and deeper levels. Second, DARC performs DCA on the family sub-MSA (as shown for the GTPases); the highest DC-scoring residue pairs (i.e., *C*_*DC*_ constraints) are displayed in available 3D-structures. Third, using Initial Cluster Analysis (ICA)(as described in Supplementary Methods), DARC identifies statistically surprising 3D-clusters of pattern residues (i.e., _*CL*_*C*_*P*_ constraints); this is shown for the Gna1 family, which exhibits a very high degree of clustering. Finally, using ICA, DARC identifies any significant correspondence among 3D contacts, DC-pairs and BPPS patterns (i.e., _*3D*_*C*_*DC*_, _*DC*_*C*_*P*_, and _*3D*_*C*_*P*_ constraints).

Given a (typically very large) multiple sequence alignment (MSA) with a specified sequence as a query, DARC hierarchically partitions the MSA into one or more query-related subgroups, each defined by a pattern that most distinguishes that subgroup’s sequences from other, closely related sequences. Such patterns presumably are due to constraints (denoted as *C*_*P*_) imposed on residues determining the functional specificity of proteins within the query’s lineage (i.e., the query’s family, subfamily, etc.). The root of the hierarchy is defined by a pattern distinguishing the entire superfamily from unrelated proteins. DARC also performs DCA^48,54^ to predict structural contacts based on pairwise constraints (denoted as *C*_*DC*_), measured as a direct coupling (DC)-score between each pair of columns within the query family sub-alignment. DARC estimates the statistical significance (_*3D*_*S*_*DC*_) of the correlation between DC-scores and 3D contacts (_*3D*_*C*_*DC*_). Viewing direct couplings as functional constraints, _*3D*_*S*_*DC*_ serves as a measure of the degree to which a given 3D structure is in a functionally relevant conformation. Likewise, for the correlation between pattern residues and DC-scores (_*DC*_*C*_*P*_) and between pattern residues and 3D-contacts (_*3D*_*C*_*P*_), DARC computes statistical significance scores _*DC*_*S*_*P*_ and _*3D*_*S*_*P*_, respectively. An insignificant value for _*DC*_*S*_*P*_ suggests that *C*_*DC*_ and *C*_*P*_ are complementary, so that a joint analysis may provide deeper biological insight. DARC likewise computes a statistical significance score (_*CL*_*S*_*P*_) for constraints (_*CL*_*C*_*P*_) tending to cluster pattern residues together spatially. To help identify determinants of protein functional specificity, DARC highlights within sequence alignments and available structures those residues subject to the strongest of each type of constraint. A DARC workflow diagram is shown in Fig. 1. Detailed descriptions of DARC algorithms and statistical models are provided as Supplementary Information.

### Identification and alignment of AAA+ sequences

We used MAPGAPS^55^ with a curated hierarchical MSA of AAA+ NTPases as the query to search the NCBI January 10^th^, 2018 release of the NCBI nr, and the April 8, 2016 releases of the env_nr, and translated EST databases^56^ to obtain over a million multiply aligned AAA+ sequences. We removed fragments (i.e., those with >25% deletions) and all but one among those sharing ≥95% sequence identity, yielding an MSA of 474,040 AAA+ modules. However, all (non-fragment) sequences corresponding to known structures detected by MAPGAPS were retained in the MSA. To check for reproducibility, we repeated the search and our analysis using the February 20, 2019 release of the nr database and the same env_nr and EST databases, yielding an MSA of 533,844 AAA+ subunits.

### *E. coli* DNA clamp loader assays

Equilibrium β clamp binding and opening assays and calculations of corresponding equilibrium constants, equilibrium DNA binding assays, steady state ATP hydrolysis assays, and thermal stability assays were performed as described in Supplementary Information.

## Results

### Bacterial clamp loader determinants of functional specificity

**Figure 2:**
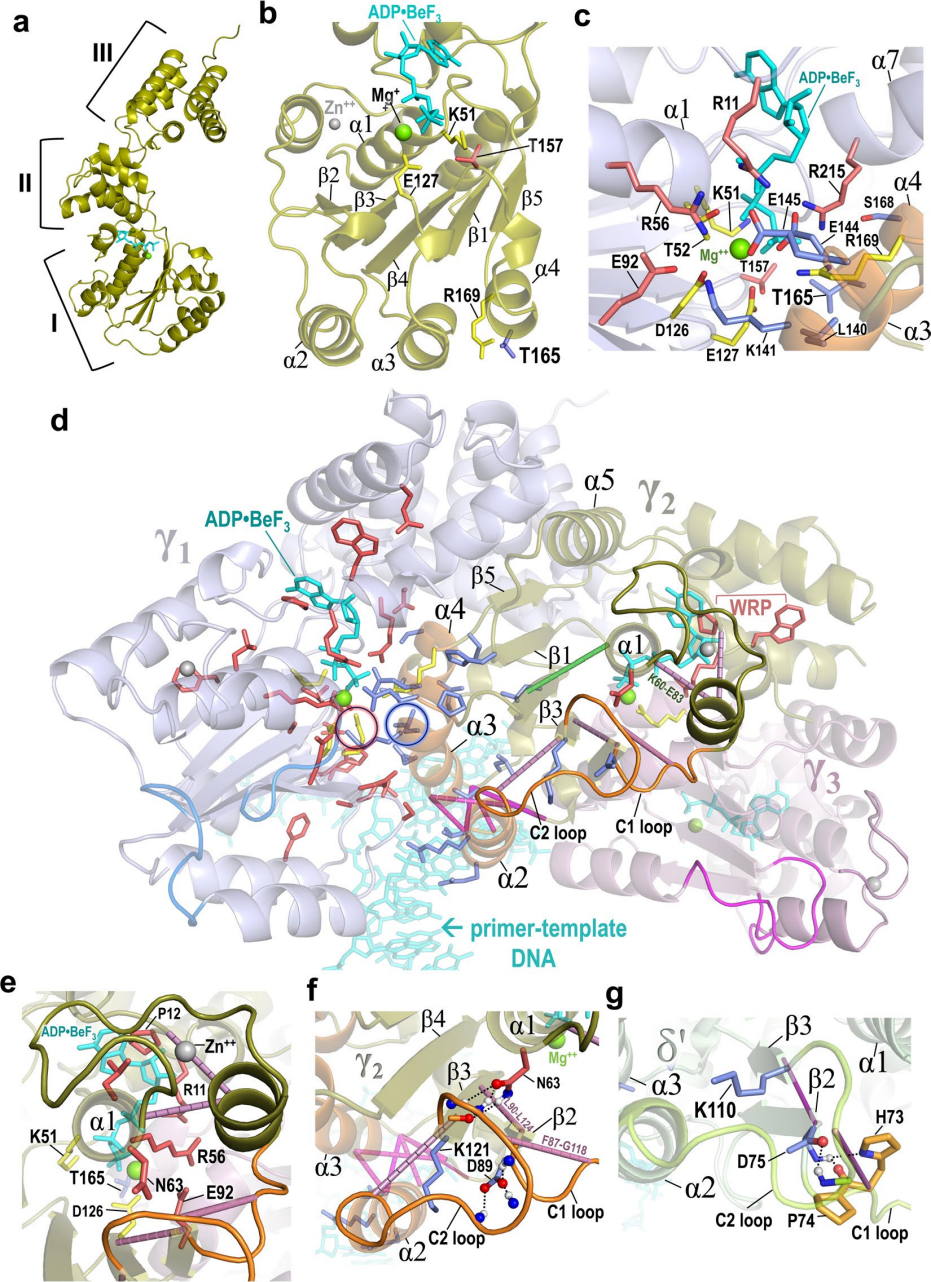
Constraints shown within the *E. coli* DNA clamp loader complex bound to primer DNA and an ATP analog (pdb_id: 3glf). **(a)** The γ-subunit. An AAA+ module^1^, which consists of an ATPase domain (I) and a three-helix bundle (II), is followed by a collar domain (III) that conjoins the five subunits. The γ_2_ subunit is shown; δ and δ’ are inactive but have similar architectures. **(b)** Domain I of γ_2_ showing Walker A (K51), Walker B (E127), and sensor 1 (T157; red) residues associated with ATP binding and hydrolysis, the trans-acting “R-finger” (R169) (also present in δ’ and most AAA + proteins) and T165 (blue), which was mutated to valine. **(c**–**g**) DARC-defined pattern residues and top DC-scoring pairs. **(c)** Interface between the γ_1_ ATP binding site and the γ_2_ helices α3 and α4. Likely hydrogen or ionic bond forming oxygen and nitrogen atoms are shown in red and blue, respectively. **(d)** Top-scoring clamp-loader-specific DC-pairs and γ- and γ/δ’-pattern residues. The γ-residues cluster around the catalytic base E127-γ_1_ (circled in red) (_*CL*_*S*_*P*_: *p* = 1.8 × 10^−9^). The γ/δ’-residues cluster around L140-γ_2_ (circled in blue) (_*CL*_*S*_*P*_: *p* = 6.7 × 10^−10^). Color scheme: Backbones of γ_1_, γ_2_ and γ_3_: *blue*, *yellow*, and *red*, respectively. Putative β-clamp binding loops C1 and C2 in γ_1_, γ_2_, and γ_3_, *marine blue*, *orange*, and *pink*, respectively. Helices α2, α3, and α4 of γ_2_, *orange*. Walker A and B catalytic residues in γ_1_ and the trans-acting R-fingers in γ_2_ and γ_3_, *yellow*; γ/δ’-residues, *blue*; γ-residues, *red*. Rods denoting high DC-scoring, clamp-loader-specific residue pairs: between the α2 and α3 N-termini, *magenta*; linked to the C1 or C2 loops, *purple*; between the α1 helix and the β4 strand, *green*; DNA and ATP analog ADP•BeF_3_, *cyan*; Zn++, *gray*; Mg++, *green*. **(e)** γ-residues near the α_1_ helix, the N-terminus of which coordinates with ATP. **(f)** K121-γ and the C1 and C2 loops proposed to bind to the β-clamp. **(g)** Region in δ’ corresponding to that shown for γ_2_ in panel (**f**); orange residues are distinctive of δ’. For clarity, DC-pairs between the α2 and α3 N-termini are not shown.

**Figure 3:**
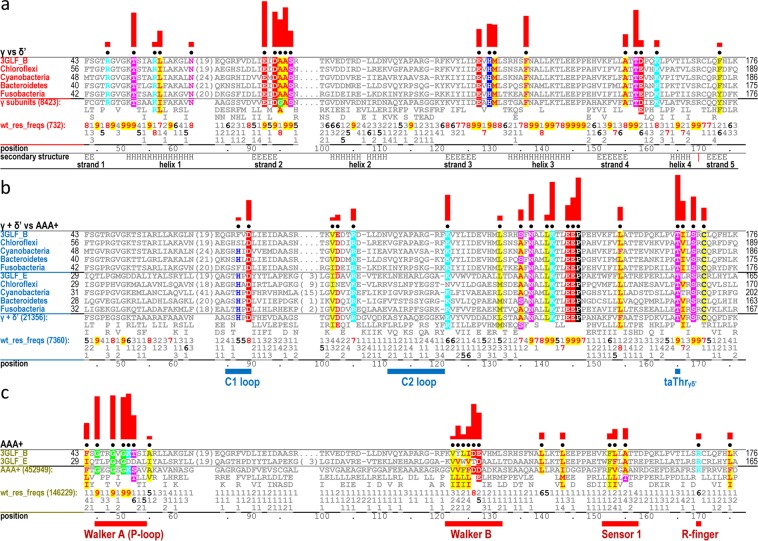
Bacterial DNA clamp loader contrast alignments. BPPS-generated alignments highlighting residues distinctive of the AAA+ superfamily, of the γ/δ’ subgroup, and of γ but not δ’. Residues are highlighted to indicate amino acid biochemical properties based on the following color code: red font with yellow highlight, non-polar (AVILMWFY); blue font with yellow highlight, cysteine (C); red, acidic (DE); cyan, basic (KR); magenta, polar (STNQ); green, glycine (G); blue, histidine (H); black, proline (P). Non-conserved positions and non-pattern residues are shown in gray font. The leftmost columns are colored the same as the residue sidechains in Fig. 2 and give the phylum for each sequence except for the two (proteobacterial) *E. coli* proteins used as queries, which are denoted by their pdb identifiers. (For more extensive alignments and for NCBI sequence identifiers, see Fig. S1.) The heights of the red bars above each highlighted column estimate the selective pressure imposed on pattern residues at that position using a semi-logarithmic scale. Directly below the representative aligned sequences, the characteristic residues at each position in the full alignment are shown and, directly below these, corresponding frequencies (after weighting for sequence redundancy) are given in integer tenths. A ‘7’, for example, indicates that the corresponding residue occurs in 70–80% of the sequences in the alignment. **(a)** Contrast alignment highlighting residues distinguishing γ from δ’. Below this the residue positions for the *E. coli* γ subunit are given and below these are predicted secondary structure elements (symbol: H, helix; E, strand) and their designations. Secondary structure assignments were calculated for the γ subunit using DSSP^85^. **(b)** Contrast alignment highlighting residues distinguishing γ and δ’ (top and bottom five sequences, respectively) from other AAA+ modules. The positions listed at the bottom again correspond to the *E. coli* γ subunit. Below these are indicated the putative clamp binding C1 and C2 loops and the taThr_γδ’_, which was mutated to valine. **(c)** Contrast alignment highlighting those residues most distinctive of the AAA+ superfamily. Below this, the locations of motifs characteristic of AAA+ ATPases are indicated.

Bacterial DNA clamps are composed of two identical *β* subunits of DNA polymerase III^57^ that encircle DNA and bind to DNA polymerase to prevent premature dissociation during replication^58–60^. The ATP-bound bacterial clamp loader binds to and opens the *β* clamp at a homodimeric interface and, upon association with primed DNA, undergoes ATP hydrolysis to dissociate from both the clamp and DNA, thereby loading the clamp onto DNA^61–63^. The minimal clamp loader complex is comprised of five AAA+ subunits—one δ, three γ and one δ’—arranged semi-circularly in the order δ-γ_1_-γ_2_-γ_3_-δ’^64^. Only γ is an active ATPase, though both γ and δ’ functionally influence an adjacent γ subunit. Figure 2a,b show the structural features of the γ subunit. The coordinated conformational changes required for clamp loading depend on interactions within and between these subunits and with ATP, the *β* clamp, and DNA^61–63^. DARC associates two sets of pattern residues with clamp loader functional specificity: Residues conserved in γ but not in δ’ (termed γ-residues) (Fig. 3a) and residues conserved in γ and δ’ but not in other AAA+ proteins (termed γ/δ’-residues) (Fig. 3b). Among the 14 structures available for the *E. coli* clamp loader complex, DARC assigns the highest significance overall (_*3D*_*S*_*DC*_ + _*3D*_*S*_*P*_) to the structure of the complex bound to primer DNA and an ATP analog (pdb_id: 3glf)^65^ (Table S1). Figure 2c–g show the locations of pattern residues and of the highest DC-scoring clamp-loader-specific residue pairs within this structure.

### Both γ/δ’- and γ-residues cluster around the catalytic base

Within the DNA + ATP bound complex (pdb: 3glf), the γ/δ’-residues, six of which interact in trans with an adjacent γ active site (Fig. 2c), tend to cluster structurally around L140-γ/L129-δ’ (Fig. 2d) with high significance (_*CL*_*S*_*P*_: *p* = 6.7 × 10^−10^). L140-γ/L129-δ’ contacts two AAA+ catalytic residues: the catalytic base in the adjacent γ subunit (E127-γ) and a trans-acting arginine (R-)finger^1^ in the same subunit (R169-γ/R158-δ’). L140-γ/L129-δ’ also packs up against T165-γ/T154-δ’ (Fig. 2c), the most distinctive γ/δ’-residue (Fig. 3b), which, for the reasons given below, is termed the “γ/δ’-trans-acting threonine” (*ta*Thr_γδ’_) and which 99% of other AAA+ proteins lack. The γ-residues (Fig. 3a), many of which interact with γ/δ’-residues in the adjacent subunit (Fig. 2c,d), likewise cluster around the catalytic base E127-γ with high significance (_*CL*_*S*_*P*_: *p* = 1.8 × 10^−9^). Hence, the catalytic base is a focal point of both γ- and γ/δ’-residues. Most of the γ/δ’-residues occur within or contact (in cis) either the α4 helix, which contains both the *ta*Thr_γδ’_ and the R-finger, or the α2 and α3 helices, the N-terminal ends of which interact with the negatively charged phosphate backbone of DNA via their positive dipole moments^65^.

### High DC-scoring pairs and γ/δ’-residues associated with DNA binding

Among the 20 highest DC-scoring residue pairs within domain I (Table 1), four couple adjacent regions to the α1 helix, the N-terminus of which interacts with phosphate groups of ATP and harbors the Walker A lysine residue K51; only one of the four pairs is clamp loader specific (green rod in Fig. 2d). Remarkably, the N-terminal ends of helices α2 and α3, which bind DNA, are joined together by six of the top DC-scoring pairs (magenta rods in Fig. 2d,f), at least five of which are clamp loader specific (Table 1; Fig. S2). In *E. coli* γ, all six pairs involve residues able to form hydrogen-bonding interactions with DNA, namely K100-γ, H134-γ, R98-γ, S132- γ, and T99-γ (pdb_id: 3glf)^65^. Several γ/δ’-residues occur near this end of the α2 helix, including R105-γ/R94-δ’, which can also form a hydrogen bonding interaction with DNA^65^. Three γ/δ’-residues within the α3 helix form inter-subunit ionic bonds with four γ-residues near the active site: E144-γ/E133-δ’ with R11-γ and R215-γ, E145-γ/E134-δ’ with R56-γ, and K141-γ/K130-δ’ with E92-γ (Fig. 2c); both R11-γ and R215-γ (the sensor 2 arginine) interact with ATP phosphate groups (Fig. 2c). Together, these interactions may allosterically link ATP binding to DNA binding or ATP hydrolysis to DNA release.

**Table 1:** The 20 highest DC-scoring residue pairs within domain I of the γ subunit (residues 42-176) based on subsampling of the γ/δ’ subalignment.

rank	*E. coli* γ pair		Description	color	% sampled^a^ among top:				% in top 20^b^	
	site 1	site 2		in Fig. 2	20	10	5	2	AAA^+^	BG
1	I54	L175	α1 to loop connecting domains I & II	not shown	100	100	94	65	99	100
3	L62	K151	α1 to β4	green	100	97	74	37		
10	L28	L58	α1 to α5	not shown	71	25	3.6	0.1	1.3	0.2
15	V18	I54	α1 to loop interacting with adenine of ATP	“	37	5.5			4.7	2.4
13^c^	V18	L25	α5 to loop interacting with adenine of ATP	“	50	6.9	0.3			0.9
2	T99	S132	links DNA-binding ends of α2 and α3	magenta	100	100	92	50		
5	V101	H134	“	“	99	86	45	9.4		
8	T99	H134	“	“	100	86	24	0.9		
9^c^	K100	S132	“	“	84	34	4.4	0.1		
18	R98	S132	“	“	33	4.0				
19	V101	S132	“	“	22	1.8	0.1			0.1
4^c^	F43	L175	β1 to loop connecting domains I & II	not shown	100	94	67	25	97	99
7	L90	L124	links β3 to β2; adjacent to D89-γ/D75-δ’	purple	97	77	34	6.4	44	73
6	K60	E83	α1 to C1 loop attached end of short helix	purple	99	86	36	5.3		
11^c^	Q13	E83	link to C1 loop attached end of short helix	“	64	13	0.5			
12	F87	G118	links the C1 and C2 loops	“	63	11	0.2			
17	V111	Y123	Links the C2 loop to β2	“	33	3.4	0.2		0.1	
14	G45	K51	conserved (γ) vs degenerate (δ’) Walker A	not shown	42	2.6	0.1		88	69
16^c^	V128	L162	links Walker B to α4 helix N-terminus	not shown	34	6.1	0.8			
20	S44	Q172	Links strands β1 and β5	not shown	22	1.8			35	11

^a^For subsampling, each of 1000 randomly drawn samples of 1,000 aligned sequences from the γ/δ’ subMSA (23,139 sequences) were used to compute DC-scores. The 4 columns give the percentage of samplings for which the column pair in each row was among the top 20,10, 5, or 2 highest scoring out of 20,517 DC-pairs.

^b^Based on 1,000 subsamples of 2,500 sequences drawn either from the full (622,021 sequence) AAA+ MSA or from the (580,241 sequence) clamp loader ‘background’ (BG) MSA (i.e., the AAA+ MSA without clamp loader proteins); pairs that fail to appear at least once in these analyses are treated as clamp loader specific.

^c^The observed residue pair occurs less frequently in the γ/δ’ subMSA than expected by chance and thus fails to contribute to the DC-score.

### Structural features associated with clamp binding

DNA clamp loader AAA+ subunits (including eukaryotic, archaeal, and bacteriophage clamp loaders) all conserve a lysine residue (K121-γ/K110-δ’ in Fig. 2f,g) at the N-terminal end of the β3 strand^66^, at the other end of which is the catalytic base E127-γ. This lysine residue shows up as a γ/δ’-residue because it is absent from essentially all non-clamp loader AAA+ proteins. Its positively charged sidechain is positioned to interact with the C-terminal negative dipole moment of the DNA-binding α2 helix, which connects to the β3 strand via a loop (termed here the C2 loop) predicted to bind to the β clamp based on homology to clamp-bound structures of both eukaryotic (pdb_id: 1sxj)^67^ and bacteriophage DNA clamp loaders (pdb_id: 3u5z, 3u60, 3u61)^68^. Because the clamp loader lysine is at the C-terminal end of the C2 loop (Fig. 2f), an ionic interaction with the C-terminal end of α2 would form a bridge connecting both ends of the C2 loop—perhaps thereby forming a conformation favoring clamp binding or release. Another γ/δ’-residue D89-γ/D75-δ’ occurs at the C-terminal end of another loop (termed the C1 loop), which similarly is predicted to bind to the clamp, and which is attached to the N-terminal end of the β2-strand; β2 is structurally adjacent to β3, which harbors K121-γ. D89-γ is sequence adjacent to L90-γ, which forms a high DC-scoring pair with β3 and is positioned to form hydrogen bonds with three backbone nitrogen atoms of the C2 loop; the C2 loop is also linked to the C1 loop by another DC-pair (F87-G118-γ in Fig. 2f and H73-G107-δ’ in Fig. 2g). The C1 loop connects, via a short helix, to the zinc binding insertion characteristic of bacterial clamp loader subunits, but not other clamp loaders. A high DC-scoring pair (K60-E83-γ; Table 1, Fig. 2d) couples this short helix to the α1 helix, the N-terminal end of which interacts with ATP. The γ-residue N63-γ occurs at the C-terminal end of the α1 helix, where it may form hydrogen bonds with backbone atoms of the C2 loop (Fig. 2f), and thus may play a role in clamp binding or in (ATP-hydrolysis-coupled) clamp release. An association between N63-γ and ATP hydrolysis is suggested by the absence of this residue from many (inactive) δ’ subunits (Figs. 3, S1a), which instead more often conserve a histidine (H73-δ’) and a proline (P74-δ’) preceding D75-δ’ (Fig. 2g). Together these C1- and C2-loop-associated features may form allosteric pathways involving ATP, the β-clamp, and DNA.

### Mutagenesis of the *ta*Thr_γδ’_ hydroxyl to a methyl group

The *ta*Thr_γδ’_ most distinguishes γ and δ’ from other AAA+ proteins (Fig. 3b). It is near the trans-acting R-finger in the same subunit and is about the same distance from the γ-phosphate of ATP as is the cis-acting sensor 1 threonine (T157-γ), which is involved in ATP hydrolysis in Hsp104^69^ and in coupling hydrolysis to restructuring of σ^54^-RNA polymerase in PspF^70^. Thus *ta*Thr_γδ’_ may sense the presence of the γ phosphate of ATP, modulate ATP hydrolysis, or help channel ATP binding or hydrolysis into conformational changes. Among the γ/δ’-residues, it is closest (in 3glf) to the center of the γ/δ’ cluster—that is, to L140-γ/L129-δ’, which contacts the adjacent γ-subunit catalytic base E127-γ at the center of the γ-residue cluster. In principle, the *ta*Thr_γδ’_ hydroxyl group could form a hydrogen bond (either directly or indirectly via a water molecule) with the catalytic base, with the R-finger, or with ATP. To investigate the possible role of this hydroxyl group, we mutated *ta*Thr_γδ’_ within δ’ and γ to a valine, which merely changes the hydroxyl to a methyl group—thereby avoiding the confounding conformational changes that more severe mutations might introduce. Activities were measured for each step in the clamp loading reaction for three different mutant complexes: one with three T165V-γ mutations; one with a T154V-δ’ mutation; and one with all four mutations. Here, we term these the γ-, δ’- and γ/δ’- mutants or mutations, respectively.

### The trans-acting hydroxyl groups facilitate clamp binding and opening synergistically

**Figure 4:**
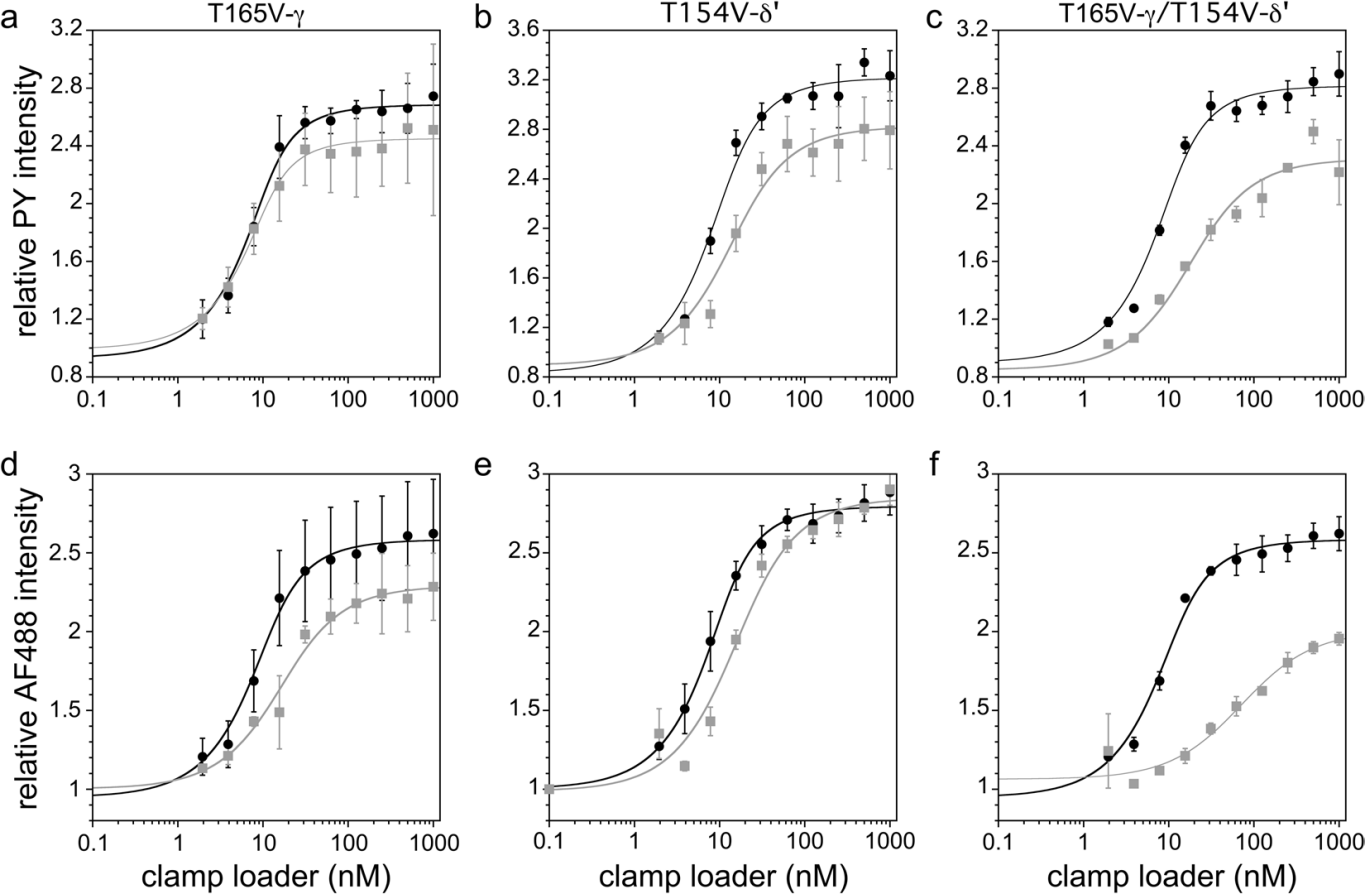
Equilibrium clamp binding and clamp opening for wild type versus mutant clamp loaders. The binding or opening activity of a clamp loader mutant was measured side-by-side with the wild-type (wt) clamp loader in triplicate. The average values and standard deviations are plotted. These titration data were fit to Equation 3 in *Supplementary Methods* to calculate apparent dissociation constants (*K*_*d,app*_) in binding assays and apparent opening constants (*K*_*op,app*_) in opening assays. The apparent equilibrium constants are a function of both the equilibrium constants for the initial binding step (*K*_*d*_) and the subsequent opening step (*K*_*op*_) for the two-step reaction. Because of this, the value of *K*_*d,app*_ will be smaller than *K*_*op,app*_ unless the value for *K*_*op*_ is much less than 1 as for the wt clamp loader. **(a-c)** Clamp binding assays contained 0.5 mM ATP, 10 nM β-PY, and wt (black circles) or mutant clamp loaders (grey squares). PY fluorescence increases when a clamp loader binds the clamp. **(a)** Plot for γ-mutant. *K*_*d,app*_ was 2.1 ± 0.4 nM for wt and 2.0 ± 0.6 nM for mutant. **(b)** Plot for δ’-mutant. *K*_*d,app*_ was 3.8 ± 1.2 nM for wt and 8.4 ± 1.2 nM for mutant. **(c)** Plot for γδ’-mutant. *K*_*d,app*_ was 2.7 ± 0.4 nM for wt to 19.3 ± 1.5 nM for mutant. **(d–f)** In clamp opening assays, AF488 fluorescence was measured when γ-clamp loader was added to β-AF488_2_. Clamp opening separates the two fluorophores and increases fluorescence. Assays contained 0.5 mM ATP, 10 nM β-AF488_2_, and wt (black circles) or mutant γ complexes (grey squares). **(d)** Plot for γ-mutant. *K*_*op,app*_ was 3.0 ± 0.6 nM for wt and 10.3 ± 2.9 nM for mutant. **(e)** Plot for δ’-mutant. *K*_*op,app*_ was 2.7 ± 0.08 nM for wt and 10.4 ± 2.1 nM for mutant. **(f)** Plot for γ**/**δ’ -mutant. *K*_*op,app*_ was 3.3 ± 0.6 nM for wt and 52.0 ± 6.8 nM for mutant.

We investigated the hydroxyl group’s role on clamp binding affinity using a fluorescence intensity-based assay^71^. To monitor the binding reaction, Glu299 of the β-clamp was mutated to Cys and covalently labeled with pyrene (PY) maleimide. Because Cys299 is located on the face of the clamp where the γ complex binds, the bound and unbound states of the clamp can be distinguished by the change in the fluorescence intensity of the environmentally sensitive probe. The γ-mutations did not affect binding of the clamp loader to the β-clamp as judged by *K*_*d,app*_ values (Fig. 4a). The *K*_*d,app*_ increased by 2 fold for δ’ mutation (Fig. 4b) and 7 fold for the γ/δ’-mutations (Fig. 4c), indicating that mutating both γ and δ’ has a synergistic effect. Moreover, the absolute PY intensity for the γ/δ’-mutant was about 75% that for wt, suggesting that mutation of the trans-acting hydroxyl group might be affecting clamp opening: Due to different environmental effects on PY, a clamp loader bound to a closed clamp fluoresces differently than one bound to an open clamp^72^.

We investigated the hydroxyl group’s role on clamp opening using an assay based on self-quenching by neighboring fluorophores. The *β* clamp was covalently labeled with AF488 on two cysteine residues, one on each side of the dimer interface, but both on the same side of the clamp. Upon clamp opening, there is an increase in AF488 fluorescence due to relief of self-quenching^73,74^ (Fig. 4d–f). The clamp opening reaction is at least a two-step reaction that consists of an initial binding step to form a closed clamp loader-clamp complex followed by a clamp opening reaction to form an open clamp loader-clamp complex (Equation 4 in *Supplementary Methods*). The relative fluorescence intensity at saturating clamp loader concentrations provides information about the relative population of clamp loader-clamp complexes in an open conformation, and the dependence of fluorescence increase on the γ-clamp loader concentration provides information about the binding affinity. The γ-mutant exhibits both a smaller increase in fluorescence intensity, 80% of that of the wt clamp loader, and a 3-fold increase in *K*_*op,app*_, the apparent clamp binding/opening constant (Fig. 4d). The population of open clamps appears unaffected for the δ’-mutant, as the increase in fluorescence intensity was comparable with the wild type clamp loader, though *K*_*op,app*_ increased 3 fold (Fig. 4e). The combined γ/δ’-mutant has the largest defect in clamp binding/opening: the fluorescence intensity at saturating concentrations was 60% that of the wt clamp loader and the *K*_*op,app*_ increased 15 fold (Fig. 4f). Together, these results show that the γ/δ’-mutation negatively affects β-clamp binding and opening. The smaller proportion of open clamps is consistent with the decreased fluorescence intensity for the γ/δ’-mutant in the β-PY binding assay.

### The trans-acting hydroxyl facilitates DNA binding

**Figure 5:**
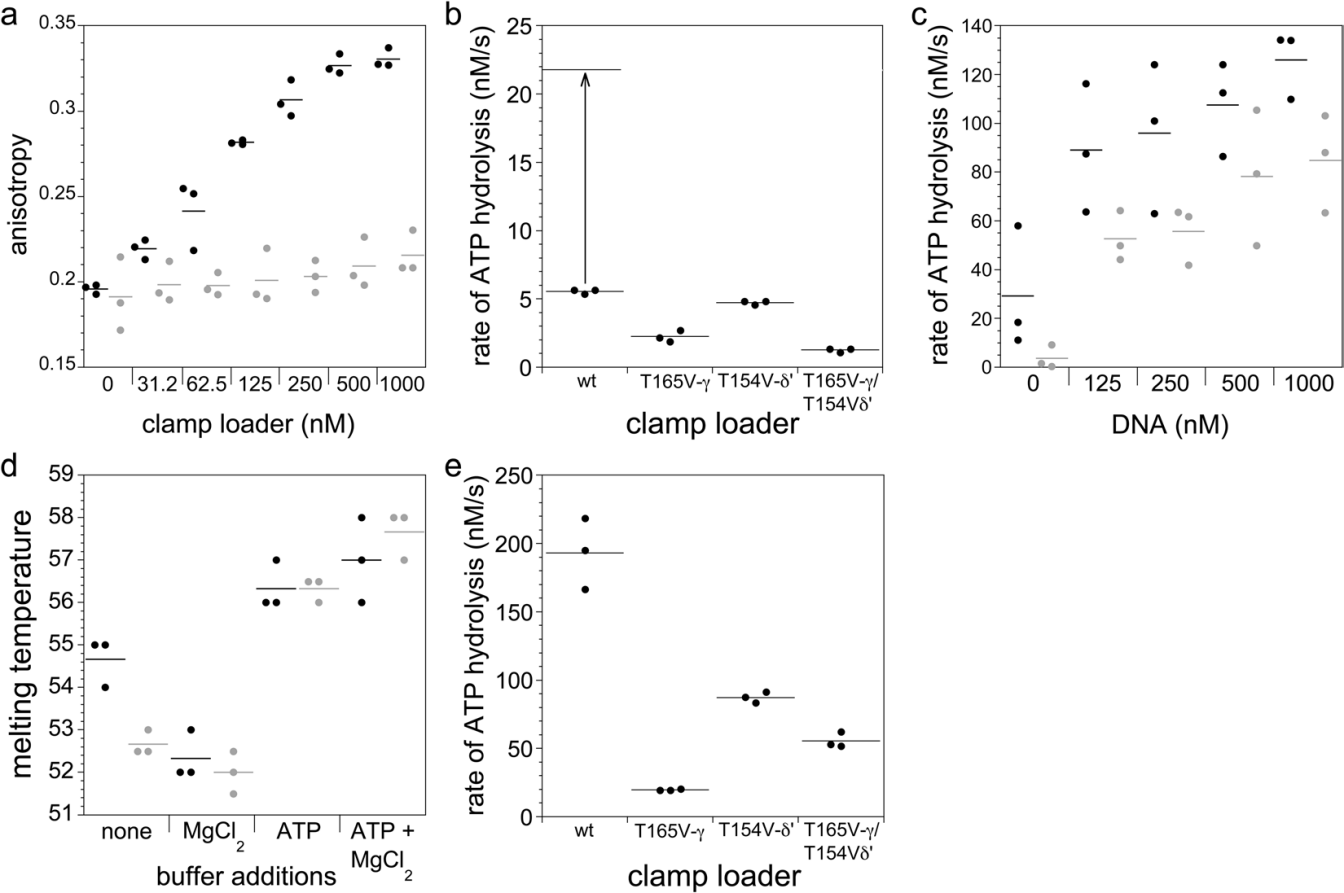
DNA binding, ATP hydrolysis and ATP binding affinities of mutant clamp loaders. Assays were performed as described in *Supplementary Methods*. For each panel, individual data points from three independent experiments are shown with the horizontal line representing the mean value. **(a)** Equilibrium binding to X-rhodamine-labeled primed template DNA by wild type (wt) and γ/δ’-mutant complexes (black and grey points, respectively) was measured by fluorescence anisotropy. Assays contained 50 nM DNA and 0.5 mM ATPγS. ATPγS was included instead of ATP to inhibit the clamp loader’s DNA-dependent ATPase activity and thereby facilitate equilibrium DNA binding. **(b)** Rates of ATP hydrolysis by wt and mutant clamp loaders were measured under *V*_*max*_ conditions (*K*_*m*_ for wt is 9.3 µM ATP^81^) using a saturating concentration of 1 mM ATP in the absence of the β-clamp. The concentration of the wt clamp loader (50 nM) was 4-fold lower than the mutants (200 nM) so that measured rates would be the same order of magnitude. Concentration-adjusted rates for the wt clamp loader are shown above the data measured at 50 nM. Values of *k*_*cat*_ were 0.111 ± 0.003 for wt, and 0.011 ± 0.002, 0.024 ± 0.001, and 0.0062 ± 0.0008 s^−1^ for γ-, δ’-, and γ/δ’-mutants, respectively. **(c)** Rates of DNA-dependent ATP hydrolysis were measured for the wt (black points) and the γ**/**δ’-mutant complex (grey points) in assays containing 0.5 mM ATP, 250 nM clamp loader, and primed template DNA. **(d)** ATP and MgCl_2_ binding to wt (black points) and γ**/**δ’-mutant clamp loader (grey points) was measured by differential scanning fluorimetry. Thermal stability (*T*_*m*_ values) were measured in assays containing the clamp loader only, the clamp loader and MgCl_2_, the clamp loader and ATP, or the clamp loader, ATP and MgCl_2_. **(e)** ATP hydrolysis was measured in steady-state clamp loading assays for the wt and γ**/**δ’-mutant clamp loaders. Assays contained 50 nM wt or mutant clamp loader, 200 nM β-clamp, 500 nM DNA, and 1 mM ATP.

We investigated the hydroxyl group’s role in loading of the clamp onto single-strand/double-strand DNA junctions, where polymerization begins, using a fluorescence anisotropy-based assay. A primed DNA template labeled with X-rhodamine (RhX) at the 5′ template end exhibits faster rotational dynamics and consequently a small anisotropy value when free in solution than when bound to clamp loader^75^. In anisotropy assays, polarized emission of RhX was measured with increasing concentrations of clamp loader (Fig. 5a). To measure equilibrium DNA binding, non-hydrolyzable ATPγS was used in place of ATP to block DNA-dependent ATP hydrolysis. The wild-type clamp loader exhibited a robust increase in anisotropy, but the γ/δ’-mutant gave only a small increase in anisotropy at high DNA concentrations. The apparent dissociation constant, *K*_*d,app*_, was 80 ± 18 nM for the wt complex, whereas the *K*_*d,app*_ for the γ/δ’-mutant was too high to determine experimentally. Thus, elimination of the trans-acting hydroxyl group severely affects ATP-dependent DNA binding activity in assays with ATPγS.

### The trans-acting hydroxyl contributes to ATP hydrolysis

We investigated the hydroxyl group’s role in ATP hydrolysis using a coupled enzyme assay, in which each mole of ADP produced is coupled to the oxidation of one mole of NADH to NAD^+^ ^76^ (Fig. S3). The value of *k*_*cat*_ for the γ/δ’-mutant was reduced the most, by a factor of about 18 (Fig. 5b). Given that ATP hydrolysis is stimulated by DNA, the DNA-dependent ATPase activity was also measured (Fig. 5c). With increasing DNA concentration, the rate of ATP hydrolysis increased for both wt and γ/δ’-mutant complexes reaching 126 ± 7 and 85 ± 8 nM/s, respectively, in the presence of 1 μM DNA. Hence, DNA rescues the ATP hydrolysis activity of the γ/δ’-mutant leading to less than a 2-fold difference in rate from the wt at DNA concentrations ≥125 nM. The γ/δ’ mutant’s lower apparent DNA binding activity as measured in anisotropy assays (Fig. 5a), which substituted the non-hydrolyzable ATP analog ATPγS for ATP, versus these ATPase assays may be due to its inability, when bound to ATPγS, to induce ATP-dependent conformational changes that increase binding affinity for the β clamp and DNA^71,77,78^.

### Eliminating the trans-acting hydroxyl does not affect ATP binding

Mutant defects in ATP hydrolysis and ATP-dependent ligand binding could be due to defects in ATP binding. To test this, ATP binding affinities were assayed (Fig. 5d) using differential scanning fluorimetry (DSF) for the wt and the γ/δ’-mutant clamp loaders, which showed the largest differences in each of the previous assays. Because ligand binding generally increases protein thermal stability, DSF is often used as a measure of ligand binding^79^. When solutions contained clamp loader only, *T*_*m*_ values were 54.7 ± 0.6 °C for the wt and 52.7 ± 0.3 °C for the γ/δ’-mutant, indicating that the mutant was inherently less stable than wt. Divalent magnesium is required for coordination of the triphosphate in the ATP binding site, and addition of 8 mM magnesium chloride (MgCl_2_) decreased the *T*_*m*_ values for both clamp loaders: to 52.3 ± 0.6 °C and 52.0 ± 0.5 °C for the wt and mutant, respectively. Addition of MgCl_2_ to the clamp loaders also resulted in the appearance of a second peak in the denaturation curve at 60 °C for both clamp loaders suggesting that two different conformational states may be present. Addition of ATP only to clamp loaders increased *T*_*m*_ values to 56.3 ± 0.6 °C for the wt and 56.3 ± 0.3 for the mutant indicating ATP stabilizes the complex. Finally, addition of both ATP and MgCl_2_ gave the largest increase in thermal stability with T_m_ values of 57.0 ± 1.0 °C for wt and 57.7 ± 0.6 °C for the mutant. In the presence of both ATP and Mg^2+^, the wild-type and mutant clamp loader show the same thermal stability indicating that the γ/δ’-mutant is binding ATP, and that deficiencies in ATP-dependent ligand interactions and ATP hydrolysis are due to intrinsic defects in those activities.

### Overall effect of the hydroxyl group on clamp loading activity

The experiments above measured each of the individual clamp loader-ligand interactions needed for DNA clamp loading, including ATP binding and hydrolysis, clamp binding and opening, and DNA binding. Mutation of the trans-acting hydroxyl to a methyl group affected each of these clamp loader-ligand interactions except for ATP binding. To determine how these deficits would affect the overall clamp loading activity, ATP hydrolysis activities of the wt and mutant clamp loaders were measured in steady-state clamp loading assays. When a clamp is loaded onto DNA, an ATP molecule at each of the binding sites in the clamp loader is hydrolyzed^80,81^, and thus ATP hydrolysis will report on clamp loading. As expected for the wt clamp loader, ATP hydrolysis activity is the greatest when coupled to clamp loading and is 35-fold faster than in assays with no DNA or β-clamp. ATPase activity of the mutants is also increased relative to activity in assays with no DNA or clamp, but all the mutants are still less active than the wt clamp loader (Fig. 5e). Compared to wt, rates of ATP hydrolysis coupled to clamp loading are 10-, 2-, and 4-fold slower for the γ-, δ’-, and γ/δ’-mutants, respectively. Interestingly, the ATPase activity of the γ/δ’-mutant was rescued to some degree by addition of both the clamp and DNA and was 40 times faster than in assays without DNA and the clamp.

## Discussion

To provide mechanistic clues to protein functional specificity, DARC characterizes six types of sequence/structural constraints (*C*_*P*_, *C*_*DC*_, _*3D*_*C*_*DC*_, _*3D*_*C*_*P*_, _*DC*_*C*_*P*_, and _*CL*_*C*_*P*_) and provides corresponding statistical significance estimates. When applied to bacterial DNA clamp loaders, it identifies distinguishing features of γ and δ’ AAA+ subunits that are congruent with our current understanding of these proteins. Certain γ and γ/δ’ residues interact with the active site and cluster around the catalytic base with high significance. Other γ/δ’ residues and six high DC-scoring pairs are associated with the α2 and α3 helices’ N-termini, which interact with DNA, whereas other constraints are associated with predicted clamp binding loops.

Whether or not certain mechanistic interpretations are correct, conservation of these features across evolutionary time argues for their functional relevance. Therefore, presumably these residues allosterically channel the energy of ATP hydrolysis into coordinated conformational changes required to load the β clamp onto DNA. Note, however, that eukaryotic and archaeal DNA clamp loaders lack the features distinctive of bacterial clamp loaders and thus presumably utilize a different mechanism.

The most distinguishing γ/δ’ feature, which is therefore likely to play a key role in bacterial clamp loader functional specificity, is a threonine hydroxyl group that interacts in trans with an adjacent active site. Mutation of the hydroxyl to a methyl group in both the γ and δ’ subunits leads to a decrease in clamp binding and opening, ATP hydrolysis and DNA binding, indicating that the hydroxyl group contributes to both ATP-dependent ligand binding and ATP hydrolysis. Although DNA stimulates ATP hydrolysis^82,83^, clamp loaders slowly hydrolyze ATP in the absence of DNA, and the γ/δ’ mutant’s DNA-independent ATPase activity is 18-fold lower than for the wild-type. This is consistent with a role for the hydroxyl group in ATP hydrolysis, as was previously hypothesized^84^. Notably, DNA and the clamp rescue γ/δ’-mutant ATPase activity perhaps due to ligand-dependent conformational changes that bring catalytic residues into the optimal geometry for ATP hydrolysis.

Because essentially all other AAA+ ATPases hydrolyze ATP without this threonine and because γ retains the common AAA+ catalytic residues, the hydroxyl group may participate in hydrolysis in a specific manner conducive to bacterial clamp loading. It may assist key steps in the clamp loader reaction by forming a hydrogen bond (perhaps via a water molecule) with ATP or with one or two residues surrounding the active site. Upon interaction of DNA with the N-termini of the (γ and δ′) α2 and α3 helices, the network of γ- and γ/δ’-residues and of high-scoring DC-pairs may allosterically mediate conformational changes involved in the clamp loading reaction that, among other effects, may cause the *ta*Thr_γδ’_ residue to form or disrupt such a hydrogen bond. In any case, the reduction of all ATP-dependent activities in the γ/δ’-mutant shows that the trans-acting hydroxyl group plays a key role in clamp loader functional specificity.

By combining BPPS with DCA, DARC identifies key features that either approach alone would have overlooked. For example, DCA of all AAA+ proteins or of all non-clamp loader AAA+ proteins fails to identify the bacterial-clamp-loader-specific residue pairs that were revealed though DCA of the γ/δ’ subgroup defined by BPPS (Table 1 and Fig. S2). The DCA and BPPS analyses likewise synergize with DARC estimation of _*3D*_*C*_*DC*_, _*3D*_*C*_*P*_, _*DC*_*C*_*P*_, and _*CL*_*C*_*P*_ constraints, which revealed, for example, that both the γ- and the γ/δ’-residues cluster around the catalytic base with high significance. Hence, this study illustrates DARC’s general utility for investigating multifaceted aspects of protein functional specificity.

## Supplementary information

Supplementary Information

## Data Availability

Data generated or analyzed during this study, if not included in this article or as Supplementary Information, are available at www.igs.umaryland.edu/labs/neuwald/software/darc/.
