# Supplementary material for: Deep Analysis of Residue Constraints (DARC): identifying determinants of protein functional specificity
Source: Sci Rep. 2020 Feb 3;10:1691. doi: 10.1038/s41598-019-55118-6 (PMC6997377; doi:10.1038/s41598-019-55118-6)
Supplement: Supplementary file 1 — Supplementary Information [file 41598_2019_55118_MOESM1_ESM.pdf]

<u>Supplementary Information:</u> Deep Analysis of Residue Constraints (DARC): identifying determinants of protein functional specificity

Farzaneh Tondnevis[1], Elizabeth E. Dudenhausen[1], Andrew M. Miller[1], Robert McKenna[1], Stephen F. Altschul[2], Linda B. Bloom[1*] and Andrew F. Neuwald[3*]

[1]Biochemistry and Molecular Biology, University of Florida, PO BOX 100245, Gainesville, Florida, 32610, United States
[2]National Center for Biotechnology Information, National Library of Medicine, National Institutes of Health, Building 38A, 8600 Rockville Pike, Bethesda, MD 20894, USA
[3]Institute for Genome Sciences and Department of Biochemistry & Molecular Biology, University of Maryland School of Medicine, 670 W. Baltimore Steet, Baltimore, MD 21201
*e-mail: lbloom@ufl.edu; aneuwald@som.umaryland.edu

1. Methods

DARC. For this study, DARC used as input: (i) an MSA of AAA+ proteins; (ii) the *E. coli* δ' or γ sequence as the query to seed the analysis of γ/δ' or γ subunits, respectively; and (iii) corresponding 3D structural coordinates. DARC executes the following: **Step 1.** Run Bayesian Partitioning with Pattern Selection (BPPS)[1-5] to define within the MSA the functionally divergent subgroups to which the query sequence belongs based on pattern residues distinguishing the members of each subgroup from other, closely related sequences. **Step 2.** Use the sub-MSA corresponding to the query's family to compute DCA-scores; this generates a DCA score output file in PSICOV format[6]. **Step 3**. For available structures, determine the statistical significance of the concurrence between DCA scores and 3D contacts (${}_{3D}S_{DC}$) and between BPPS scores and 3D contacts (${}_{3D}S_{P}$). **Step 4**. Identify statistically significant 3D clusters of BPPS-defined residues (${}_{CL}S_{p}$). **Step 5**. Use PyMOL (http://www.pymol.org/) to visualize pattern residues and directly coupled residue pairs within protein structures. Structural coordinate files were obtained from the RCSB protein data bank (PDB)[7]. The PDB identifiers for proteins examined here are: 3glf, 3glg, 3glh, 3gli, 1a5t, and 1jr3. Hydrogen atoms were added to these files using the Reduce program[8] version 3.3; this may also be done using the PyMOL *h_add* command.

Bayesian Partitioning with Pattern Selection (BPPS). Given a typically very large multiple sequence alignment (MSA), denoted here as $\mathbf{X}$, BPPS applies Markov chain Monte Carlo (MCMC) sampling to articulate a superfamily into a set of hierarchically nested partitions corresponding to a tree. Each subtree *h*, which may consist of only a single node, when attached to the root corresponds to a family, and, in general, when attached to a parent node corresponds to a child subgroup. The sampler defines each subgroup, also denoted by *h*, based on residue patterns distinguishing subgroup members from

sequences assigned to other nodes in the parent subtree. For instance, a simple pattern for subgroup *h* might consist of $\{\text{V,I,L}\}$, $\{\text{D,E}\}$, and $\{\text{F,Y}\}$ at column positions 3, 10 and 23, respectively. BPPS favors assignment of those sequences to subtree *h* conserving a pattern that is not conserved in sequences assigned to other nodes in the parent subtree. Hence, BPPS favors assignment to each parent node those sequences conserving the parent node's pattern but lacking each of the descendent nodes' patterns. For a non-root node *n* in the hierarchy this process defines a 'contrast' alignment (as in **Fig. S1**) divided into foreground and background sequences, corresponding respectively to the subtree rooted at *n* and to the rest of the subtree rooted at the parent of *n*. MCMC sampling is used to determine the number and arrangement of the nodes in the tree, the sequences belonging to each node, the pattern positions for each subgroup, and the conserved residues at each pattern position. The sampler favors convergence on a hierarchy where the pattern defining the partitioning for each node best distinguishes its foreground from its background. BPPS weights sequences, as in PSI-BLAST[9,10], to avoid modeling conserved patterns merely due to sequence redundancy.

For the ensuing discussion, we define the following: For vectors $\mathbf{v} = (v_1,...,v_\ell)^T$ and $\mathbf{w} = (w_1,...,w_\ell)^T$, $\mathbf{v}/\mathbf{w} = (v_1/w_1,...,v_\ell/w_\ell)^T$, $\mathbf{v}+\mathbf{w} = (v_1+w_1,...,v_\ell+w_\ell)^T$, $\log\mathbf{v} = (\log v_1,...,\log v_\ell)^T$, $|\mathbf{v}|$ is the sum over vector elements, and $\langle \mathbf{v},\mathbf{w} \rangle$ denotes the inner product of **v** and **w** and is equivalent, as applied here, to the dot product $\mathbf{v}\cdot\mathbf{w} = \sum_{i=1}^{\ell} v_i w_i$. Given an *N* node hierarchy $\mathbf{H}$, we define **S** as a vector of *N* disjoint sets, such that $\mathbf{S}_n$ contains the sequences assigned to node *n*, and $\mathbf{H}$ is defined as a vector of tri-partitions of node indices $1 \le n \le N$, such that $\mathbf{H}_h \equiv \langle H_h^+, H_h^-, H_h^o \rangle$ specifies subtree *h*'s foreground, background and "non-participating" nodes, respectively. We define node *n* = 1 as the root and *h* = 1 as the superfamily tree (i.e., $H_1^+ = \{n \,|\, 1 < n \le N\}$), for which the background set ($H_1^- = \{0\}$) consists of a single node (*n* = 0) for unrelated sequences (denoted as $\mathbf{S}_0$). The remaining $\mathbf{H}_h$ are configured hierarchically starting from the root, such that: H_h^+ specifies the nodes in subtree *h*; H_h^- specifies the nodes in the tree rooted at the parent node of *h* but absent from H_h^+; and H_h^o specifies nodes in neither H_h^+ nor H_h^-. To ensure that $\mathbf{H}$ corresponds to a tree, we require that each H_h^+, other than H_1^+, is a proper subset of only one other $H_{h'}^+$ (i.e., $\forall h : h > 1 \rightarrow \exists! h' : H_h^+ \subset H_{h'}^+$) and that H_h^- consist of nodes in $H_{h'}^+$ that are not in H_h^+ $\left(\text{i.e., } H_h^- = H_{h'}^+ - H_h^+\right)$.

BPPS defines a prior on **H** that depends only on N and positive parameter ν and that assumes a maximum number of nodes $N_{\max}$, so that

$$p(\mathbf{H}) = \frac{p(N)}{a_N}, \text{ where } p(N) = \begin{cases} \nu^{N-1} \cdot (1-\nu)/(1-\nu^{N_{\max}}) & \text{if } \nu \neq 1 \\ 1/N_{\max} & \text{if } \nu = 1 \end{cases},$$

and where a_N, the number of unlabeled, unordered rooted trees with N nodes, is defined recursively as:

$$a_N = \begin{cases} 1 & \text{if } N = 1 \\ \sum_{\substack{j_1 + 2j_2 + \ldots + (N-1)j_{N-1} \\ = N-1}} \prod_{k=1}^{N-1} \binom{a_k + j_k - 1}{j_k} & \text{if } N > 1 \end{cases}$$

with j_k being the number of subtrees with k nodes[11]. Computation suggests that the growth of a_N is $\mathrm{O}(2.96^N)$. By default, $N_{\max} = 500$ and $\nu = 1$ so that $p(\mathbf{H}) = (a_N N_{\max})^{-1}$, which corresponds to a uniform prior where every size tree (up to $N_{\max}$) is equally likely. Setting $\nu > 1$ or $\nu < 1$ favors hierarchies with more or fewer nodes, respectively. Note, however, that adding nodes when unjustified by the data is disfavored regardless of $p(\mathbf{H})$ due to the nature of our Bayesian formulation.

Let σ_n be the prior for a sequence assigned to node n. Given N, the prior for **S** is then given by $p(\mathbf{S}) = \prod_{n=0}^{N} \sigma_n^{|S_n|}$. By default, we choose a prior for the rejected sequence node of $\sigma_0 = 0.5$ and for other nodes $\sigma_n = (1-\sigma_0) \cdot N^{-1}$ uniformly.

Given **H** and **S**, foreground pattern residue sets are denoted by $\mathbf{A}$, where $A_{h,c} \neq \varnothing$ for each pattern column position c in subgroup h, and $A_{h,c} = \varnothing$ for non-pattern positions. The pattern residue sets $\mathbf{A}_h$ are constrained by a foreground consensus sequence for subgroup h, denoted as $\mathbf{y}_h$, and by the requirement that the residues in each set be functionally similar, as were defined by a detailed analysis of amino acid Dirichlet mixture components[12]. For example, if a tryptophan residue occurs at position c of the consensus for H_h^+, then $y_{h,c} = \mathrm{W}$ and

$$\mathbb{A}(\mathrm{W}) \equiv \{\{\mathrm{W}\}, \{\mathrm{W,F}\}, \{\mathrm{W,Y}\}, \{\mathrm{W,F,Y}\}\} \text{ and } A_{h,c} \in \mathbb{A}(\mathrm{W}) \cup \{\varnothing\},$$

where $\mathbb{A}(r)$ denotes the allowed pattern residue sets for consensus residue *r*. We define prior probabilities for $\mathbf{A}$ as $p(\mathbf{A}) = \prod_{h=1}^{N}\prod_{c=1}^{C}\rho_{A_{h,c}}$ (product categorical distributions), where, when $A_{h,c} = \varnothing$, $\rho_{A_{h,c}} = q_{\varnothing}$ (0.999 by default) and otherwise $\rho_{A_{h,c}} = \kappa \cdot \frac{q^{|A_{h,c}|}}{\|A_{h,c}\|}$. Here, κ is a constant chosen so that the priors for pattern positions sum to $1 - q_{\varnothing}$; $0 < q < 1$ (and 0.5 by default) is a tuning parameter with smaller values yielding higher aggregate priors for the class of "functional" residue sets of smaller cardinality $|A_{h,c}|$; and $\|A_{h,c}\|$, defined as the number of possible residue sets of cardinality $|A_{h,c}|$, functions to distribute prior probabilities uniformly among these sets. For example, if $y_{h,c} = \mathrm{W}$ and $A_{h,c} = \{\mathrm{W,F}\}$, then $\|A_{h,c}\| = 2$ because, in this case, there are two possible sets of cardinality 2.

Since we are interested only in whether a residue in column *c* of a sequence *s* (i.e., $x_{s,c}$) is functional or non-functional, we introduce the variable $\boldsymbol{\chi}$, where $\boldsymbol{\chi}_{h,s,c} = (1,0)^{\mathrm{T}}$ and $\boldsymbol{\chi}_{h,s,c} = (0,1)^{\mathrm{T}}$ imply that, for subtree *h*, $x_{s,c}$ corresponds to a 'functional' and a 'non-functional' pseudo-residue, respectively. Since $A_{h,c} = \varnothing$ at non-pattern columns, corresponding residues are all non-functional. Gaps are also treated as non-functional.

Given **H**, **S** and **A**, let $\boldsymbol{\theta}_{h,c}$ be the 2-dimensional vector specifying the observed functional and non-functional pseudo-residue background frequencies for column *c* of subtree *h*, and let $\boldsymbol{\Theta}$ be the matrix of all $\boldsymbol{\theta}_{h,c}$. Let $\boldsymbol{\theta}_{h,c}^{(\alpha_h)} \equiv (1-\alpha_h)\boldsymbol{\theta}_{h,c} + \alpha_h (1,0)^{\mathrm{T}}$ model the foreground composition where $1-\boldsymbol{\alpha}$ specifies the fraction of background 'contamination' at pattern positions in the foreground. The prior probability density for α_h is defined by a beta distribution

$$p(\alpha_h) = \frac{\Gamma(a_{h,0} + b_{h,0})}{\Gamma(a_{h,0})\Gamma(b_{h,0})}\alpha_h^{a_{h,0}-1}(1-\alpha_h)^{b_{h,0}-1},$$

where $a_{h,0}$ and $b_{h,0}$ are functional and non-functional pseudo-counts, respectively, and where by default $a_{h,0} = b_{h,0} = 1$. The prior probability density for $\boldsymbol{\theta}_{h,c}$ is defined by a product Beta distribution:

$$p(\boldsymbol{\Theta}) = \prod_{h=1}^{N}\prod_{c=1}^{C}\frac{\Gamma(a+b)}{\Gamma(a)\Gamma(b)}\theta_{h,c,1}^{a-1}\theta_{h,c,2}^{b-1},$$

where *C* is the number of columns in the MSA, and $a = b = 1$ by default.

Conditional on **H**, $\boldsymbol{\chi}$ and **S**, let $\xi_{h,c}$ denote the inferred number of functional pseudo-residues in column *c* of subtree *h* that are *not* due to background contamination. Then, conditional on α_h and $\boldsymbol{\theta}_{h,c}$,

$$\xi_{h.c} \left| \alpha_h , \boldsymbol{\theta}_h \right. \sim \text{Binom}\left(Nf_{h,c}, \frac{\alpha_h}{\alpha_h + (1-\alpha_h)\theta_{h,c,1}} \right),$$

where $\theta_{h,c,1}$ and $Nf_{h,c}$ are, respectively, the background frequency and the total foreground number (with background contamination included) of the pattern-matching pseudo-residues in column *c* for subtree *h*. Conditional on **H**, $\boldsymbol{\chi}$, **S**, $\boldsymbol{\xi}_h \equiv \left(\xi_{h,1},...,\xi_{h,C}\right)^{\mathrm{T}}$, and $Nf_{h.} = \sum_c Nf_{hc}$, the posterior distribution of α_h is

$$\left[\alpha_h \left| \boldsymbol{\xi}_h \right.\right] \propto \alpha_h^{|\boldsymbol{\xi}_h| + a_{h,0} - 1} \left(1-\alpha_h\right)^{Nf_{h.} - |\xi_h| + b_{h,0} - 1} \sim \text{Beta}\left(|\boldsymbol{\xi}_h| + a_{h,0}, Nf_h - |\boldsymbol{\xi}_h| + b_{h,0} \right).$$

The conditional distribution for $\boldsymbol{\theta}_{h,c}$ is:

$$\boldsymbol{\theta}_{h,c} \left| \mathbf{H}, \mathbf{S}, \boldsymbol{\xi}_h, \boldsymbol{\chi}_h \right. \sim \text{Beta}\left(\sum_{n \in H_h^-} \sum_{s \in S_n} \chi_{h,s,c,1} + Nf_{h,c} - \xi_{h,c} + \psi_1, \sum_{n \in H_h^- \cup H_h^+} \sum_{s \in S_n} \chi_{h,s,c,2} + \psi_2 \right),$$

where $\boldsymbol{\psi} \equiv (a,b)^{\mathrm{T}}$ specifies pseudo-counts with $\boldsymbol{\psi} = (1,1)^{\mathrm{T}}$ by default.

The sampler infers $\mathbf{H}, \mathbf{S}, \mathbf{A}, \boldsymbol{\alpha},$ and $\boldsymbol{\Theta}$ from $\mathbf{X}$, which defines an MSA. Given these variables, the logarithm of the joint probability distribution[3] is defined as:

$$\log P\left(\mathbf{X}, \mathbf{H}, \mathbf{S}, \mathbf{A}, \boldsymbol{\alpha}, \boldsymbol{\Theta}\right) = \log P\left(\mathbf{X} \left| \mathbf{H}, \mathbf{S}, \mathbf{A}, \boldsymbol{\alpha}, \boldsymbol{\Theta} \right.\right) + \log p(\mathbf{H}) + \log p(\mathbf{S}) + \log p(\mathbf{A}) + \log p(\boldsymbol{\alpha}) + \log p(\boldsymbol{\Theta}) \quad (1)$$

where, assuming statistical independence among subtrees (but see below),

$$\log P\left(\mathbf{X} \left| \mathbf{H}, \mathbf{S}, \mathbf{A}, \boldsymbol{\alpha}, \boldsymbol{\Theta} \right.\right) = \sum_{h=1}^{N} \left(\sum_{n \in H_h^+ \cup H_h^-} \sum_{s \in S_n} \sum_{c=1}^{C} \left\langle \log \boldsymbol{\theta}_{h,c}, \boldsymbol{\chi}_{h,s,c} \right\rangle + \sum_{n \in H_h^+} \sum_{s \in S_n} \sum_{c=1}^{C} \boldsymbol{I}_{A_{h,j}} \left\langle \log \frac{\boldsymbol{\theta}_{h,c}^{(\alpha_h)}}{\boldsymbol{\theta}_{h,c}}, \boldsymbol{\chi}_{h,s,c} \right\rangle \right) \quad (2)$$

and where $\boldsymbol{I}_{A_{h,c}} \equiv \begin{cases} 0, & \text{if} \quad A_{h,c} = \varnothing \\ 1, & \text{if} \quad A_{h,c} \neq \varnothing \end{cases}$. Note that for non-pattern positions $\boldsymbol{\theta}_{h,c} = (0,1)^{\mathrm{T}}$ and $\chi_{h,s,c} = (0,1)^{\mathrm{T}}$ so that $\left\langle \log \boldsymbol{\theta}_{h,c}, x_{sc} \right\rangle = \log 0 \cdot 0 + \log 1 \cdot 1 = 0$; hence, these positions contribute nothing to the posterior probability. However, because the configuration of each subtree constrains to some degree the possible configurations of other subtrees above or below it in the hierarchy, our independence assumption is invalid. These constraints reduce the probabilities for some states to zero, so that the probabilities assigned to the remaining, reachable states will sum to less than 1, and our formulation is therefore conservative (i.e., computed probabilities are smaller than they should be). This also occurs due to other imposed constraints, such as placing an upper bound on the number of pattern positions or on the depth

of the hierarchy, or requiring that a minimum number of sequences be assigned to each node. Nevertheless, in searching for an optimum, Equation 1 is valid as an objective function, its use here.

BPPS sampling strategies. Conditioned on fixed **H**, BPPS samples over **S** and **A** by iteratively applying the following. For each sequence *s*, let *n* be its assigned node and remove *s* from S_n. Then, sample *s* to a new node n' with probability proportional to $P\left(\mathbf{X},\mathbf{H},\mathbf{S},\mathbf{A},\boldsymbol{\alpha},\boldsymbol{\Theta}\middle|s\in S_{n'}\right)$ after having updated $\boldsymbol{\Theta}$ and **α**. Likewise, for each column position *c* in each subtree *h*, remove the pattern set $A_{h,c}$ and sample in a new pattern set $A'_{h,c}\in\mathbb{A}(\mathrm{y}_{h,c})\cup\{\varnothing\}$ with probability proportional to $P\left(\mathbf{X},\mathbf{H},\mathbf{S},\mathbf{A},\boldsymbol{\alpha},\boldsymbol{\Theta}\middle|A_{h,c}=A'_{h,c}\right)$ after having updated **χ**, $\boldsymbol{\Theta}$ and $\boldsymbol{\alpha}$. However, if the number of pattern positions for a given subtree *h* is greater than a specified maximum $C_{\max}$ (25 by default), reduce the number down to $C_{\max}$ by removing the lowest probability pattern positions.

The BPPS sampler is initialized by setting $N=1$ with $\mathbf{H}_1=\left\langle H_h^+=\{1\},H_h^-=\{0\},H_h^o=\varnothing\right\rangle$, and assigning all sequences to the root node ($n=1$) with $C_{\max}$ pattern positions for subtree $h=1$ and with background pseudo-residue frequencies at each position (the $\boldsymbol{\theta}_{0,c}$) derived from the overall residue frequencies for the entire MSA. At this stage, sampling over **S** merely involves iteratively assigning sequences either to the foreground (S_1) or to the background (S_0), where the background represents unrelated sequences inadvertently included in the alignment. Sampling over **A** generally tweaks pattern assignments slightly due to removal of unrelated sequences. This provides a good starting point to speed up convergence with essentially no risk of getting trapped in a suboptimal state. Convergence is defined by a cycle of sampling over **S** and **A** that fails to improve upon the best configuration found thus far, as defined by the log-probability (Equation 1). BPPS saves the best configuration for the final output.

After convergence with $N = 1$ nodes, a child node may be added to the root node, as follows. First, some of the sequences assigned to the root are reassigned to the child node by selecting a subset of sequences that are more similar to each other than they are to the remaining sequences. Selections are based on similarity to the query, when one is designated, and on similarity to an arbitrary sequence otherwise. Next, BPPS samples for a few cycles over **S** and **A**, as described above, to search for a configuration that improves upon the previous hierarchy based on Equation 1. If the hierarchy fails to improve and a query has not been provided, several other candidate queries may be selected in turn until either an improved state is found or until a prespecified number of attempts are tried. If the hierarchy is improved, BPPS further enlarges and rearranges the evolving hierarchy **H** by adding more leaf nodes

and by deleting, inserting or moving nodes using this same basic strategy. To avoid excessively complex hierarchies, BPPS requires that each leaf node contain a minimum number of sequences (50 by default); those that do not are pruned. After convergence, the sampler applies simulated annealing[13] to 'drop into' a more nearly optimal configuration.

When DARC applies BPPS, it focuses on the query's lineage within the superfamily hierarchy by first defining the query's family based on residues that most distinguish family members from other superfamily members. Next, DARC seeks to recursively define, in a similar manner, the query's subfamily, and other subgroups further down the query's lineage to a prespecified maximum depth. For the analysis here, using a MSA of 463,471 AAA+ proteins and the *E. coli* δ' clamp loader subunit as the query, BPPS identified those residues that most distinguish both γ and δ' from other AAA+ proteins (**Fig. S1c**). Using the γ/δ' sub-MSA and the *E. coli* γ subunit as a query, BPPS identified those residues that most distinguish γ from δ' (**Fig. S1e**). DARC saves a BPPS checkpoint file that can later be used to initiate a deeper analysis by expanding subtrees within the query's lineage.

Direct Coupling Analysis (DCA). DARC performs DCA using the algorithm implemented in the CCMpred program version 0.3.2 (https://travis-ci.org/soedinglab/CCMpred)[14], which is essentially identical to the plmDCA[15] and GREMLIN[16] algorithms and which we modified to output DCA scores in PSICOV format. Our description here follows closely the one given for CCMpred[14]. The rationale behind DCA is that, over evolutionary time, mutations at a given residue position are compensated for by mutations at interacting positions to thereby maintain structural integrity. DCA works by avoiding the confounding effect of indirect correlations due, for example, to two residues both interacting with a third residue, but not with each other. DARC used the γ + δ' sub-MSA defined by BPPS to compute the highest scoring directly coupled residue pairs (DC-pairs) listed in **Table 1**.

The CCMpred algorithm eliminates indirect interactions from an interaction network by inferring a generative model of the MSA based on a Markov Random Field (MRF). We again represent the input MSA as an *R* row × *C* column matrix **X**, where element $x_{s,c}$ corresponds to the residue in row (i.e., sequence) *s* and column *c*. The columns correspond to vertices of the MRF with single-residue emission potentials $\varepsilon_c(r)$ for amino acid residue $r \in \{1,...,20\}$ in column *c*; covariation between columns corresponds to edges of the MRF with pairwise emission potentials $\varepsilon_{c,d}(r_c, r_d)$ for residues r_c and r_d in columns *c* and *d*, respectively. In theory, one could optimize the parameters of the MRF given the MSA using as the objective function the probability:

$$P(\boldsymbol{\varepsilon}|\mathbf{X}) = \frac{1}{Z}\prod_{s=1}^{R}\prod_{c=1}^{C}\left[\exp\left(\varepsilon_c\left(x_{s,c}\right) + \sum_{\substack{d=1\\d\neq c}}^{C}\varepsilon_{c,d}\left(x_{s,c}, x_{s,d}\right)\right)\right]$$

where Z is a normalization constant to ensure that the sum over all sequences equals 1. However, because computing $P(\boldsymbol{\varepsilon}|\mathbf{X})$ is intractable for a non-trivial MSA, the following pseudo-log-likelihood is used instead as the objective function:

$$plL(\boldsymbol{\varepsilon}|\mathbf{X}) = \sum_{s=1}^{R}\sum_{c=1}^{C}\left[\varepsilon_c\left(x_{s,c}\right) + \sum_{\substack{d=1\\d\neq c}}^{C}\varepsilon_{c,d}\left(x_{s,c}, x_{s,d}\right) - \log Z_{s.c}\right]$$

$$\text{where } Z_{s,c} = \sum_{r=1}^{20}\exp\left[\varepsilon_c(r) + \sum_{\substack{d=1\\d\neq c}}^{C}\varepsilon_{c,d}\left(r, x_{s,c}\right)\right].$$

Because computation of the normalization constants $Z_{s,c}$ involve summing over only C terms, these are much faster to compute than Z for $P(\boldsymbol{\varepsilon}|\mathbf{X})$. The gradient (the vector of partial derivatives) of this pseudo-log-likelihood is given by:

$$\frac{\partial plL(\boldsymbol{\varepsilon}|\mathbf{X})}{\partial\varepsilon_{c,d}(r,r')} = \sum_{s=1}^{R}\left\{\delta_{x_{s,d},r'}\left[\delta_{x_{s,c},r} - \frac{1}{Z_{s,c}}\exp\left(\varepsilon_c(r) + \sum_{\substack{i=1\\i\neq c}}^{C}\varepsilon_{i,c}\left(r, x_{s,i}\right)\right)\right]\right\}$$
$$= \sum_{s=1}^{R}\left[\delta_{x_{s,d},r'}\right]\left[\delta_{x_{s,c},r} - p\left(x_{s,c} = r\,\middle|\left(x_{s,1},\ldots,x_{s,c-1},x_{s,c+1},\ldots,x_{s,C},V,E\right)\right)\right]$$

where $\delta_{x,y}$ is the Kronecker delta function.

In order to favor sparse solutions, we add an L_2 regularization term $R(\boldsymbol{\varepsilon})$ and maximize $plL(\boldsymbol{\varepsilon}|\mathbf{X}) - R(\boldsymbol{\varepsilon})$ using the nonlinear conjugate gradient method where

$$R(\boldsymbol{\varepsilon}) = \lambda_{\text{single}}\sum_{c=1}^{C}\left\|\varepsilon_c\right\|_2^2 + \lambda_{\text{pair}}\sum_{\substack{c,d=1\\d\neq c}}^{C}\left\|\varepsilon_{c,d}\right\|_2^2,$$

where the regularization coefficients are $\lambda_{\text{single}} = 1$; $\lambda_{\text{pair}} = 0.2 \times (L - 1)$ [16], and where $\left\|V_c\right\|_2^2$ and $\left\|E_{c,d}\right\|_2^2$ are the sum of squared residuals, which measure the discrepancy between the model and the data (with smaller values indicating a tighter fit of the model to the data).

After a successful optimization, the couplings between residue positions $K_{c,d}$ are ranked by the Frobenius norms of the edge potentials $\varepsilon_{c,d}$:

$$K_{c,d} = \sqrt{\sum_{r,r'=1}^{20} \varepsilon_{c,d}\left(r,r'\right)^2}$$

Lastly, an Average Product Correction[17] is applied to arrive at the final score:

$$\zeta_{c,d} = K_{c,d} - \frac{K_{c,\cdot} K_{\cdot,d}}{K_{\cdot,\cdot}}$$

where "." denotes averaging over the corresponding row or column and $K_{\cdot,\cdot}$ is the average over all matrix elements.

Evaluating the robustness of DCA score rankings. To determine whether different input MSAs rank DC-pairs consistently, an auxiliary subsampling routine is included as an option in DARC. For the analysis here, this routine draws from the input MSA 1,000 samples of 1,000 sequences, from each of which DCA scores are computed. Between samplings, the previously sampled sequences are replaced prior to sampling the subsequent set. The percentage of times that each residue pair was among those with the top 20, 10, 5 or 2 DC-pairs is given in Table 1. Those pairs consistently selected among the 20 top scores were included in Fig. 2 and Table 1.

Initial Cluster Analysis (ICA). To compute ${}_{CL}S_P$, ${}_{3D}S_{DC}$, ${}_{3D}S_P$, or ${}_{DC}S_P$ (denoted generically here as S) we apply Initial Cluster Analysis[18], a statistical approach to address the following question: Consider an array of 0s and 1s of length L and containing D 1s. Are some or all of the 1s significantly clustered near the start of the array, and, if so, how surprising is the most significant such clustering? To make this determination, ICA applies the Minimum Description Length (MDL) principle[19], an information theoretical regularization method for finding the best hypothesis for a given set of data.

The MDL principle defines a theory θ as a probability distribution P_θ over all possible sets of data and the description length of a data set E given a theory θ as $\mathrm{DL}\left(E|\theta\right) = -\log\left(P_\theta\left(E\right)\right)$. A model $\mathcal{M}$ is a parameterized set of theories, and the description length of E given $\mathcal{M}$ is defined as $\mathrm{DL}\left(E|\mathcal{M}\right) = \min_{\theta\in\mathfrak{M}} \mathrm{DL}\left(E|\theta\right)$. The MDL principle asserts that among multiple models to explain E, one should prefer the model $\mathcal{M}$ that minimizes $\mathrm{DL}\left(E|\mathcal{M}\right) + \mathrm{COMP}\left(\mathcal{M}\right)$, where the description length or complexity $\mathrm{COMP}\left(\mathcal{M}\right)$ is the log of the number of effectively independent theories $\mathcal{M}$ contains.

For ICA, the MDL principle determines whether the hypothesis $\mathcal{H}_1$ that the 1s cluster near the start of the sequence is better than the null hypothesis $\mathcal{H}_0$ that the 1s and 0s occur randomly.

ICA treats $\mathcal{H}_1$ as a single-parameter model, whose parameter x describes the location of a cut at a discrete point from 1 to $L-1$ along the array, thereby dividing it into an initial segment s_1 of length x, and a terminal segment s_2 of length $y = L - x$. If s_1 contains D_1 1s, and s_2 contains $D_2 = D - D_1$ 1s, assume that s_1 is generated by Bernoulli trials with maximum-likelihood probability $P_1 = D_1/x$ for a 1, and s_2 is generated by Bernoulli trials with probability $P_2 = D_2/y$ for a 1. Given a particular fixed value for x, the probability of E is $P_x(E) = P_1^{D1}(1-P_1)^{x-D_1} P_2^{D_2}(1-P_2)^{y-D_2}/Z$, where Z is a normalization constant taken over all length L sequences having D 1s. Hence the description length of E under $\mathcal{H}_1$ is $\mathrm{DL}(S|\mathcal{H}_1) = -\log(\max_x P_x(E))$. ICA computes the complexity of $\mathcal{H}_1$, as

$$\mathrm{COMP}(\mathcal{H}_1) \approx \log\left(\sqrt{D/\pi}\,\frac{L-1}{2}\right).$$

ICA treats $\mathcal{H}_0$ as a model consisting of a single Bernoulli-trial theory for generating E, with the probability of a 1 taken as $P = D/L$, and of a 0 as $Q = 1 - P$. Hence, $\mathrm{DL}(E|\mathcal{H}_0) = -\log(P^D Q^{L-D})$, which is L times the entropy of the Bernoulli trial. Because $\mathcal{H}_0$ contains only one theory, its complexity is zero. The MDL principle says that we should prefer $\mathcal{H}_1$ to $\mathcal{H}_0$ when $\mathrm{DL}(E|\mathcal{H}_1)$ + $\mathrm{COMP}(\mathcal{H}_1)$ < $\mathrm{DL}(E|\mathcal{H}_0)$. Treating each hypothesis as equally likely *a priori*, we may view the difference Δ between the two sides of this inequality as a log-odds ratio, and use the logistic function $\frac{e^{\Delta}}{1+e^{\Delta}}$ to convert this into a p-value (see p. 37 of [20]), from which $S = -\log_{10}(p)$ is defined.

DCA and 3D contacts concurrence scores ($_{3D}S_{DC}$). The $_{3D}S_{DC}$-scores apply ICA[18] to measure, as the $-\log_{10}(p)$, the statistical significance of the correspondence between pairwise structural interactions and DC scores. Given an array of residue pairs ordered by their DC scores, we ask how well it agrees with an alternative ordering based on 3D pairwise distances. More specifically, we seek to identify an optimal initial cluster of elements of the array (defined by a cut), as measured by a relevant p-value. We are given an array of L residue pairs ordered by their DC-scores. D of the pairs (denoted by '1's) are

separated within a reference 3D structure by ≤ z Å (with z = 3.5 Å by default) and L - D (denoted by '0's) are not.

We ask: what initial cluster, consisting of pairs up to and including a cut point X, contains the most surprising number d of '1's, and what is its probability of occurring by chance? (We term the d 1s in an initial cluster "left-distinguished pairs.") For L = 18 and D = 7, for example, one such array is "101101100000010001", with optimal cut point X = 7 (underlined), yielding d = 5. Since the pairs are ranked by pairwise distance, we might then represent our example array as "401603200000070005" with digits > 0 denoting the ranks of distinguished pairs. ICA ignores these ranks when choosing the optimal X, whereas we would prefer the d distinguished pairs to the left of X to have superior ranks (i.e., lower numbers) than those to the right.

To generalize ICA to exploit ranking information we incorporate a ball-in-urn model to calculate a ranking specific p-value P_b. For a specific cut point X that yields d left-distinguished pairs, we imagine first coloring red, among all D distinguished pairs, those d pairs with the smallest pairwise distances; and then recording the number R that are red among the left-distinguished pairs. Ideally, all the left-distinguished pairs will outrank the remaining distinguished pairs, yielding $R = d$, but more generally higher values of R are better; in the example of the previous paragraph, D = 7, d = 5 and R = 4. Given the null hypothesis that rankings are random, we may then use the cumulative hypergeometric distribution to calculate the probability P_b that ≥ R of the left-distinguished pairs are red:

$$P_b = \left[\sum_{i=R}^{d} \binom{d}{i}\binom{D-d}{d-i}\right] \div \binom{D}{d}.$$

This corresponds to drawing d balls from an urn containing D balls, of which d are red; note that the number of balls drawn here equals the number colored red. A low value of P_b is reported for a cut with a surprising number, among its d left-distinguished pairs, having the d smallest pairwise distances.

Before it corrects for having optimized over all possible cuts, ICA can be understood as calculating a p-value P_a for finding d distinguished pairs to the left of a cut point X. Because the calculation of P_a ignores ranking information, it will be independent of P_b, and these two p-values may therefore be combined to yield a joint p-value P_J [21-23] using the formula

$$P_J = P_a P_b \left(1 - \ln P_a P_b\right).$$

Low values of P_J may arise from low values of P_a, or P_b, or of both. P_J can provide a statistically stronger measure of the congruence of two orderings, here derived from DC scores and 3D distances,

than does P_a alone. The *p*-values *P* we report in this paper correspond to P_J, after it has been corrected for optimization over the multiple cut points *X* considered[18].

For homomeric structures, DARC assesses the correspondence, not only between DCA scores and *internal* 3D-contacts alone (e.g., labeled as 'A' for chain A), but also between DCA scores and both *internal* and adjacent subunit *interface* 3D-contacts (e.g., labeled as 'A:B' for chain A and adjacent chain B). The change in ${}_{3D}S_{DC}$ upon inclusion of interface contacts is denoted as ΔS_{DC}. High positive values for ΔS_{DC} suggest that strong selective pressures are maintaining 3D contacts between adjacent subunits. In contrast, negative values for ΔS suggest that subunit interactions may be functionally insignificant and perhaps due to a crystallographic artifact.

BPPS and DCA or 3D contact concurrence scores (${}_{DC}S_P$, ${}_{3D}S_P$). DARC provides a measure of statistical significance (based on ICA) for the concurrence between pairs of BPPS-defined residues and either the highest scoring DC pairs or the closest 3D-contacts. The overlap between BPPS and DCA[24] assessed in this way is often weak. Hence, DCA and BPPS are often complementary, so that combining both analyses often provides deeper insight into the relationship between protein structure and function.

Pattern residue 3D-clustering significance scores (${}_{CL}S_P$). DARC applies ICA to estimate constraints tending to cluster BPPS-defined residues structurally[25]. The *L* positions of the ICA array correspond to the *L* residues within a 3D structure of the DARC query protein or of other proteins belonging to the query family. The 1s in the array correspond to a fixed number of BPPS-defined pattern residues and the 0s to the remaining residues. DARC orders array elements based on their 3D distance from a starting residue. It then determines the most significant 3D-cluster of these residues among a nested set of clusters, each centered on the starting residue. This is performed, starting with each of the BPPS-defined residues in turn, and the highest scoring 3D-cluster among these is reported along with the starting residue and the corresponding ${}_{CL}S_P$-score. The ${}_{CL}S_P$ score measures the significance of the intersection between a 3D cluster and the BPPS residue set. In addition to the strategy just described (termed "spherical expansion"), DARC allows either core expansion or hydrogen-bond-network expansion[25]. Core expansion sequentially adds the residue closest to a residue within the cluster's "core". This core is defined as the starting residue *R* plus all cluster residues whose distance to their k^{th} closest cluster residue is less than *R*'s distance to its kth closest cluster residue (with k=7 by default; this was selected empirically to avoid both spherical- and tentacle-shaped clusters.) In this case, the cluster typically expands less symmetrically. Hydrogen-bond-network expansion sequentially adds a residue

forming the closest sidechain-to-sidechain or sidechain-to-backbone hydrogen bond with a cluster residue.

Equilibrium β clamp binding assay. Fluorescence was measured using a QuantaMaster QM1 spectrofluorometer (Photon Technology International). Reagents were added to 3x3x5 mm quartz cuvette (Hellma 105.251-QS) to a final volume of 80 μL and data was collected at room temperature immediately after mixing. A β mutant, Glu-299 to Cys, was covalently labeled with N-(1-pyrene) maleimide at residue 299 to measure β binding by γ-complex clamp loader [26]. To selectively label Cys-299, two cysteines on the surface of the β clamp (Cys-260 and Cys-333) were mutated to serine. Pyrene was excited at 345 nm and emission was measured from 355 nm to 455 nm with a 4 nm band-pass. At each clamp loader concentration, reagents were sequentially added to the cuvette and fluorescence emission was measured for 1) buffer containing 0.5 mM ATP, 2) after addition of β^{PY}, and 3) after addition of the γ-clamp loader. Storage buffer was added instead of γ complex to generate the 0 nM γ-clamp loader point. After correcting for buffer background, the intensity of bound β^{PY} at 375 nm was divided by the intensity for free β^{PY} at each clamp loader concentration. To correct for dilution, each value was then divided by that obtained for 0 nM γ complex, setting this point to 1 and all other points are relative to this value. Data were analyzed using KaleidaGraph. Each experiment was repeated three times and average values of relative intensities at 375 nm with standard deviations (error bars) are plotted. The apparent $K_{d,app}$ was estimated by applying non-linear regression to the function

$$I_{obs} = \frac{(K_{d,app} + \gamma_c + \beta) - \sqrt{(K_{d,app} + \beta + \gamma_c)^2 - 4(\beta)(\gamma_c)}}{2(\beta)}(I_{max} - I_{min}) + I_{min} \quad (3)$$

where γ_c is total concentration of clamp loader, β is total concentration of β^{PY} and I_{max} and I_{min} are maximum and minimum emission intensities, respectively.

Equilibrium β clamp opening assay. The basic protocol for clamp opening assays is the same for β binding assays except that a different β construct is used. For opening assays, a β mutant in which Arg-103 and Ile-305 were mutated to Cys and surface Cys-260 and Cys-333 were mutated to Ser was labeled with Alexa Fluor 488 (AF488) maleimide (Invitrogen) [27]. AF488 was excited at 490 nm and emission was measured from 500 nm to 550 nm with a 3 nm band-pass. As in the β binding assay, three measurements were taken: 1) buffer containing ATP and $MgCl_2$, 2) solution after addition of β^{AF488}, and 3) solution after the addition of γ complex. Storage buffer was added instead of γ complex to generate the 0 nM γ-clamp loader point. After correcting for buffer background, the intensity of bound β^{AF488} at

517 nm was divided by the intensity for free β^{AF488} at each clamp loader concentration. To correct for dilution, each value was then divided by that obtained for 0 nM γ-complex, setting this point to 1. All other points are relative to this value. Each experiment was repeated three times and average values of relative intensities at 517 nm with standard deviations (error bars) are plotted. The apparent $K_{op,app}$ was calculated using Equation 3, with $K_{op,app}$ in place of $K_{d,app}$ and β^{AF488} concentration in place of β^{PY} concentration.

Relationship between $K_{d,app}$ and $K_{op,app}$. Because the binding and opening assays measure different species, apparent equilibrium constants calculated from these assays may differ. Apparent dissociation ($K_{d,app}$) constants measured in the β-PY binding assay and binding/opening equilibrium ($K_{op,app}$) constants measured in the β-$AF488_2$ opening assay are a function of the microscopic equilibrium constants for both the discrete binding and opening reactions

$$\gamma + \beta^c \leftrightarrows \gamma.\beta^c \leftrightarrows \gamma.\beta^o, \qquad (4)$$

where γ denotes the γ complex, β^c denotes a closed clamp, and β^o denotes an open clamp, and where the equilibrium dissociation constant for the first binding step and the equilibrium constant for the opening step are defined as $K_d = \frac{[\gamma][\beta^c]}{[\gamma.\beta^c]}$ and $K_{op} = \frac{[\gamma.\beta^c]}{[\gamma.\beta^o]}$, respectively. In the β-PY binding assay, clamp loader-clamp complexes contribute to the increase in fluorescence regardless of whether they are open or closed, whereas in the opening assay, only open complexes give an increase the observable signal. The apparent equilibrium constants measured in these assays are defined as $K_{d,app} = \frac{[\gamma][\beta^c]}{([\gamma.\beta^c] + [\gamma.\beta^o])}$ and $K_{op,app} = \frac{[\gamma][\beta^c]}{[\gamma.\beta^o]}$. Therefore, the apparent dissociation ($K_{d,app}$) and opening ($K_{op,app}$) constants differ, and can be expressed in terms of the microscopic equilibrium constants as $K_{d,app} = K_d\left(\frac{K_{op}}{K_{op}+1}\right)$ and $K_{op,app} = K_d K_{op}$. In general, the value of $K_{d,app}$ will be smaller than $K_{op,app}$ by a factor of $1/(K_{op}+1)$. However, when the value for K_{op} is much less than one, the value of $K_{d,app}$ will be about the same as the value of $K_{op,app}$. For the wild-type clamp loader, the majority of the clamp loader-clamp complexes, at least 98% [28], are in an open conformation giving a K_{op} that is significantly less than one, and the average $K_{d,app}$ (2.9 nM) and the average $K_{op,app}$ (3.0 nM) are the same within experimental error. If mutations decrease the fraction of open clamp loader-clamp complexes increasing K_{op} relative to the wt clamp loader, the $K_{d,app}$ will be smaller than $K_{op,app}$. This is the case for the T165V-γ/T154V-δ' mutant where the value of $K_{d,app}$ (19.3 nM) is smaller than $K_{op,app}$ (52.0 nM).

Anisotropy based equilibrium DNA binding assay. DNA binding was measured using a fluorescence-based anisotropy assay [29]. For these assays, a 26-nt DNA primer was annealed to a 60-nt template covalently labeled with X-rhodamine (RhX) isothiocyanate via an amino linker (5' amino modifier C6) incorporated at the 5'-end. RhX was excited at 585 nm and emission was measured at 605 nm using an 8-nm bandpass. Glan-Thompson polarizers were used to generate linearly polarized vertical and horizontal light. Polarized emission was measured for 30 s at a rate of 1 point per second, and an average value calculated from the 30 points. Data were corrected for polarization bias (G) of the detectors using Equation 5, and anisotropy (r) values were calculated using Equation 6 where I_{HV} and I_{HH} are vertically and horizontally polarized emission, respectively, measured with horizontal excitation, and I_{VV} and I_{VH} are vertically and horizontally polarized emission, respectively, measured with vertical excitation for samples containing buffer only (bkgd) or DNA (DNA).

$$G = \frac{I_{HV(DNA)} - I_{HV(bkgd)}}{I_{HH(DNA)} - I_{HH(bkgd)}} \qquad (5)$$

$$r = \frac{I_{VV(DNA)} - I_{VV(bkgd)} - G(I_{VH(DNA)} - I_{VH(bkgd)})}{I_{VV(DNA)} - I_{VV(bkgd)} + 2G(I_{VH(DNA)} - I_{VH(bkgd)})} \qquad (6)$$

Assays contained 0.5 mM ATPγS, 50 nM primed template DNA, and clamp loader concentrations ranging from 0 to 1 µM. Apparent $K_{d,app}$ values were calculated using Equation 3 with replacement of β-PY with DNA-RhX concentration.

Steady state ATP hydrolysis assay. To measure ATPase activity of the clamp loader complex, an enzyme-coupled reaction was used in which pyruvate kinase (PK) and lactate dehydrogenase (LDH) ultimately oxidize one mole of NADH to NAD+ per mole of ADP produced by the clamp loader [30,31]. Loss of NADH is measured by decrease in absorbance at 340 nm or fluorescence emission at 460 nm.

The reaction scheme for the PK/LDH-coupled assay is as followed:

(1) ATP ⇆ ADP + Pi, by the clamp loader

(2) phosphoenolpyruvate + ADP ⇆ ATP + pyruvate, by PK

(3) pyruvate + NADH + H^+ ⇆ NAD^+ + lactate, by LDH

In Fig. 5b and 5e, the ATP hydrolysis-dependent decrease in NADH concentration was measured by the decrease in absorbance at 340 nm, and in Fig. 5c, ATP hydrolysis was measured by the decrease in NADH fluorescence at 460 nm using a 2 nm bandpass. For absorbance measurements, the change in

absorbance over time is then converted to the rate of ADP production using the extinction coefficient of NAD^+ (ε_{340} = 6220 $M^{-1}cm^{-1}$) [30]. The change in absorbance at 340 nm was monitored at one second intervals in a Cary Bio-3 spectrophotometer. Each reaction mixture contained 20mM Tris-Cl pH 7.5, 50 mM NaCl, 8 mM $MgCl_2$ to which was added 1 mM ATP, 2 mM PEP (Sigma), 0.16 mM NADH (Sigma), 5 U PK (Sigma) and 8 U LDH (Sigma) (final concentrations). Unless otherwise noted both the β-clamp and primer/template DNA were added at a final concentration of 200 nM. All reactions were carried out at room temperature in a total volume of 500 µl. The reactions were initiated by the addition of γ clamp loader at concentrations given in the figure legends. For each of the γ clamp loaders assays, reactions rates were measured without the addition of either DNA, β-clamp or ATP as negative controls. For fluorescence measurements, the buffer background contained, 1 mM PEP, 0.2 mg/mL NADH, 68 units/mL PK, 99 units/mL LDH, 0.5 mM ATP and varying concentrations of p/t DNA. Reactions were started by adding 250 nM γ-clamp loader 50 seconds after the scan was started. The scan was continued for an additional 500 s and the velocity was linearly fit to calculate the slope for the decrease in fluorescence as a function of time (**Supplementary Figure S3b**). To correct for dilution effects on NADH fluorescence, a reading was taken where storage buffer was added without γ-clamp loader. To compute the rate of ATP hydrolysis in units of nM/s, a standard curve was used to measure the change in NADH fluorescence upon addition of varying concentrations of ADP (0-200 µM). The slope of a linear fit of these data was used to convert the slopes obtained from ATPase assays to the rate of ATP hydrolysis (**Supplementary Figure S3a**).

Differential scanning fluorimetry thermal stability assay: The melting temperature of the clamp loaders (T_m) were measured by an increase fluorescence intensity of a dye, SYPRO Orange, that occurs when the protein unfolds exposing hydrophobic regions to which the dye binds. The assays were run in a Bio-Rad MyiQ2 Thermocycler instrument, with temperature ranging from 30°C to 99°C and ramping at 0.5°C per step[32,33]. SYPRO Orange fluorescence was measured using FAM (485 nm) excitation and ROX (625 nm) emission filters. The rate of change of fluorescence with temperature (dRFU/dT) was recorded, and normalized by dividing the raw values of the profile by the peak dRFU/dT value. The maximum value of the first derivative (dRFU/dT) from the raw signal was used to define T_m. For DSF measurements, 2 mg/ml of γ-clamp loader (either wild type or mutant) was diluted in 20mM Tris-HCl @ pH 7.5 buffer with or without $MgCl_2$ (8mM final concentration) and with or without ATP (0.5mM final concentration). To each sample, 2.5 µl of 1% Sypro-Orange dye (no. S6651; Invitrogen) was added to

the solution for a final volume of 25 μl for each reaction. The clamp loader concentration was approximated to be 0.2 mg/ml after the dilution of the protein in Tris-HCl buffer and dye.

References:

1 Neuwald, A. F. Surveying the manifold divergence of an entire protein class for statistical clues to underlying biochemical mechanisms. *Statistical applications in genetics and molecular biology* **10**, 36 (2011).

2 Neuwald, A. F. Protein domain hierarchy Gibbs sampling strategies. *Statistical applications in genetics and molecular biology* **13**, 497-517, doi:10.1515/sagmb-2014-0008 (2014).

3 Neuwald, A. F. A Bayesian sampler for optimization of protein domain hierarchies. *J Comput Biol* **21**, 269-286, doi:10.1089/cmb.2013.0099 (2014).

4 Neuwald, A. F. & Altschul, S. F. Inference of Functionally-Relevant N-acetyltransferase Residues Based on Statistical Correlations. *PLoS Comput Biol* **12**, e1005294, doi:10.1371/journal.pcbi.1005294 (2016).

5 Neuwald, A. F., Kannan, N., Poleksic, A., Hata, N. & Liu, J. S. Ran's C-terminal, basic patch, and nucleotide exchange mechanisms in light of a canonical structure for Rab, Rho, Ras, and Ran GTPases. *Genome Res* **13**, 673-692, doi:10.1101/gr.862303 (2003).

6 Jones, D. T., Buchan, D. W., Cozzetto, D. & Pontil, M. PSICOV: precise structural contact prediction using sparse inverse covariance estimation on large multiple sequence alignments. *Bioinformatics* **28**, 184-190, doi:10.1093/bioinformatics/btr638 (2012).

7 Rose, P. W. *et al.* The RCSB Protein Data Bank: views of structural biology for basic and applied research and education. *Nucleic Acids Res* **43**, D345-356, doi:10.1093/nar/gku1214 (2015).

8 Word, J. M., Lovell, S. C., Richardson, J. S. & Richardson, D. C. Asparagine and glutamine: using hydrogen atom contacts in the choice of side-chain amide orientation. *J Mol Biol* **285**, 1735-1747. (1999).

9 Altschul, S. F. *et al.* Gapped BLAST and PSI-BLAST: a new generation of protein database search programs. *Nucleic Acids Res* **25**, 3389-3402 (1997).

10 Henikoff, S. & Henikoff, J. G. Position-based sequence weights. *J Mol Biol* **243**, 574-578 (1994).

11 Knuth, D. E. *The Art of Computer Programming*. 3rd edn, Vol. 1 386-388,395 (1997).

12 Nguyen, V. A., Boyd-Graber, J. & Altschul, S. F. Dirichlet mixtures, the Dirichlet process, and the structure of protein space. *J Comput Biol* **20**, 1-18, doi:10.1089/cmb.2012.0244 (2013).

13 Kirkpatrick, S., Gelatt, C. D. & Vecchi, M. P. Optimization by simulated annealing. *Science* **220**, 671-680 (1983).

14 Seemayer, S., Gruber, M. & Soding, J. CCMpred--fast and precise prediction of protein residue-residue contacts from correlated mutations. *Bioinformatics* **30**, 3128-3130, doi:10.1093/bioinformatics/btu500 (2014).

15 Ekeberg, M., Lövkvist, C., Lan, Y., Weigt, M. & Aurell, E. Improved contact prediction in proteins: Using pseudolikelihoods to infer Potts models. *Physical Review E* **87**, 012707, doi:10.1103/PhysRevE.87.012707 (2013).

16 Kamisetty, H., Ovchinnikov, S. & Baker, D. Assessing the utility of coevolution-based residue–residue contact predictions in a sequence- and structure-rich era. *Proceedings of the National Academy of Sciences* **110**, 15674-15679, doi:10.1073/pnas.1314045110 (2013).

17 Dunn, S. D., Wahl, L. M. & Gloor, G. B. Mutual information without the influence of phylogeny or entropy dramatically improves residue contact prediction. *Bioinformatics* **24**, 333-340, doi:10.1093/bioinformatics/btm604 (2008).

18 Altschul, S. F. & Neuwald, A. F. Initial Cluster Analysis. *J Comput Biol* **25**, 121-129, doi:10.1089/cmb.2017.0050 (2018).

19 Grunwald, P. D. *The minimum description length principle*. (MIT Press, 2007).

20 Durbin, R., Eddy, S., Krogh, A. & Mitchison, G. *Biological sequence analysis: probabilistic models of proteins and nucleic acids*. (Cambridge University Press, 1998).

21 Bailey, T. L. & Gribskov, M. Combining evidence using p-values: application to sequence homology searches. *Bioinformatics* **14**, 48-54 (1998).
22 Fisher, R. A. *Statistical Methods for Research Workers*. (Oliver and Boyd, 1954).
23 Yu, Y. K., Gertz, E. M., Agarwala, R., Schaffer, A. A. & Altschul, S. F. Retrieval accuracy, statistical significance and compositional similarity in protein sequence database searches. *Nucleic Acids Res* **34**, 5966-5973, doi:10.1093/nar/gkl731 (2006).
24 Neuwald, A. F. & Altschul, S. F. Statistical investigations of protein residue direct couplings. *PLoS Comput Biol* **14**, e1006237, doi:10.1371/journal.pcbi.1006237 (2018).
25 Neuwald, A. F., Aravind, L. & Altschul, S. F. Inferring joint sequence-structural determinants of protein functional specificity. *Elife* **7**, doi:10.7554/eLife.29880 (2018).
26 Thompson, J. A., Paschall, C. O., O'Donnell, M. & Bloom, L. B. A slow ATP-induced conformational change limits the rate of DNA binding but not the rate of beta clamp binding by the escherichia coli gamma complex clamp loader. *J Biol Chem* **284**, 32147-32157, doi:10.1074/jbc.M109.045997 (2009).
27 Paschall, C. O. *et al.* The Escherichia coli clamp loader can actively pry open the beta-sliding clamp. *J Biol Chem* **286**, 42704-42714, doi:10.1074/jbc.M111.268169 (2011).
28 Douma, L. G., Yu, K. K., England, J. K., Levitus, M. & Bloom, L. B. Mechanism of opening a sliding clamp. *Nucleic Acids Res* **45**, 10178-10189, doi:10.1093/nar/gkx665 (2017).
29 Bloom, L. B. *et al.* Dynamics of loading the beta sliding clamp of DNA polymerase III onto DNA. *J Biol Chem* **271**, 30699-30708 (1996).
30 Lindsley, J. E. Use of a real-time, coupled assay to measure the ATPase activity of DNA topoisomerase II. *Methods Mol Biol* **95**, 57-64 (2001).
31 Norby, J. G. Coupled assay of Na+,K+-ATPase activity. *Methods Enzymol* **156**, 116-119 (1988).
32 Bennett, A. *et al.* Thermal Stability as a Determinant of AAV Serotype Identity. *Mol Ther Methods Clin Dev* **6**, 171-182, doi:10.1016/j.omtm.2017.07.003 (2017).
33 Rayaprolu, V. *et al.* Comparative analysis of adeno-associated virus capsid stability and dynamics. *J Virol* **87**, 13150-13160, doi:10.1128/JVI.01415-13 (2013).

2. Figure S1.

Figure S1. DARC-generated alignments highlighting all residues conserved in γ and δ' clamp loader proteins and residues distinctive of the AAA+ superfamily, of the γ + δ' subgroup, and of γ but not δ'. These are shown using five versions of the same representative set of γ proteins (in panles a-e) and of δ' proteins (in panels a to c). Residues are highlighted to indicate amino acid biochemical properties based on the following color code: red font with yellow highlight, non-polar (AVILMWFY) ; blue font with yellow highlight, cysteine (C); red, acidic (DE); cyan, basic (KR); magenta, polar (STNQ); green, glycine (G); blue, histidine (H); black, proline (P). Non-conserved positions in panels (a) and (d) and non-pattern residues in panels (b), (c) and (e) are shown in gray font. The leftmost columns in panels (b), (c) and (e) give the NCBI sequence identifiers; these are colored the same as the residue sidechains in Figure 2 of the paper. **a**. Alignment highlighting all γ + δ' conserved residues. Those sequences above the line within the alignment correspond to representative γ proteins from the (distinct) phyla denoted in the leftmost column; the first sequence corresponds to the *E. coli* γ subunit (pdb_id: 3glfB). Those sequences below the line correspond to representative δ' proteins from distinct phyla, the first sequence of which corrsponds to the *E. coli* δ' subunit (pdb_id: 3glfE), which was used as the DARC query. The positions listed at the bottom correspond to the *E. coli* γ subunit. **b**. BPPS contrast alignment showing the same sequences as in panel (a), but highlighting only those residues most distinctive of the AAA+ superfamily. The heights of the red

bars above each highlighted column estimate the selective pressure imposed on pattern residues at that position using a semi-logarithmic scale. Directly below the aligned sequences, the characteristic AAA+ residues at each position are shown and, directly below these, corresponding frequencies are given in integer tenths. A '7', for example, indicates that the corresponding residue occurs in 70-80% of the 452,949 AAA+ sequences in the alignment. Below this is shown the residue positions and sequence of the *E. coli* γ subunit (with the Thr 165 residue that was mutated to Val highlighted in red), and shown below these are predicted secondary structure elements (symbol: H, helix; E, strand), helix and strand designations, and AAA+ structural motifs (red font) and putative clamp binding loops C1 and C2 (green font). Secondary structure assignments were calculated for the *E. coli* γ subunit using DSSP {PMID 6667333}. **c**. BPPS contrast alignment using the same format as in panel (b) to highlight those residues that most distinguish γ and δ subunits from other AAA+ proteins. **d**. DARC-generated alignment highlighting all residues conserved in γ. **e**. DARC-generated alignment highlighting residues distinguishing γ subunits from δ' subunits. A few of these are conserved in other catalytically active AAA+ ATPases; see panel (b).

a

γ + δ' AAA+ domains:

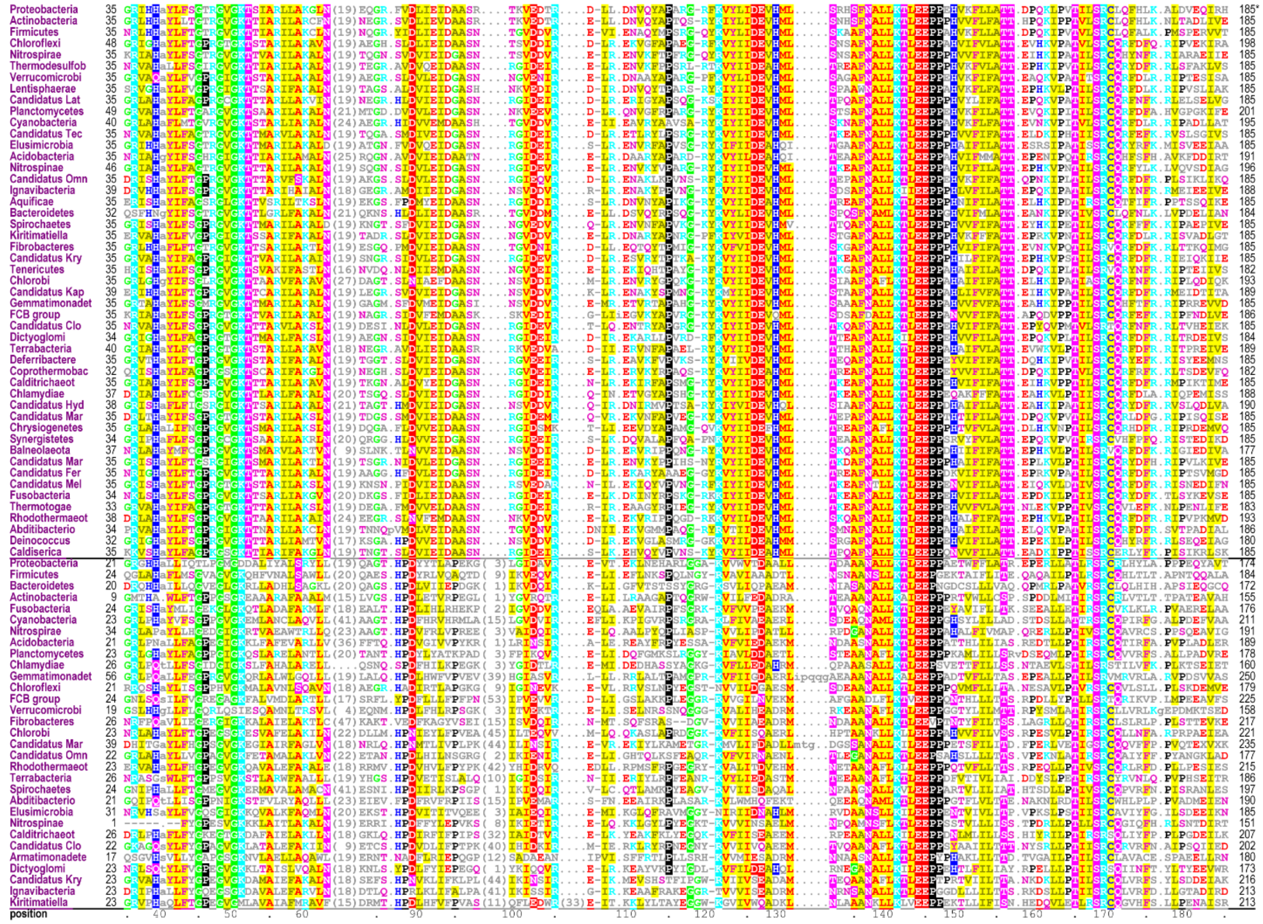

Figure S1 (cont.)

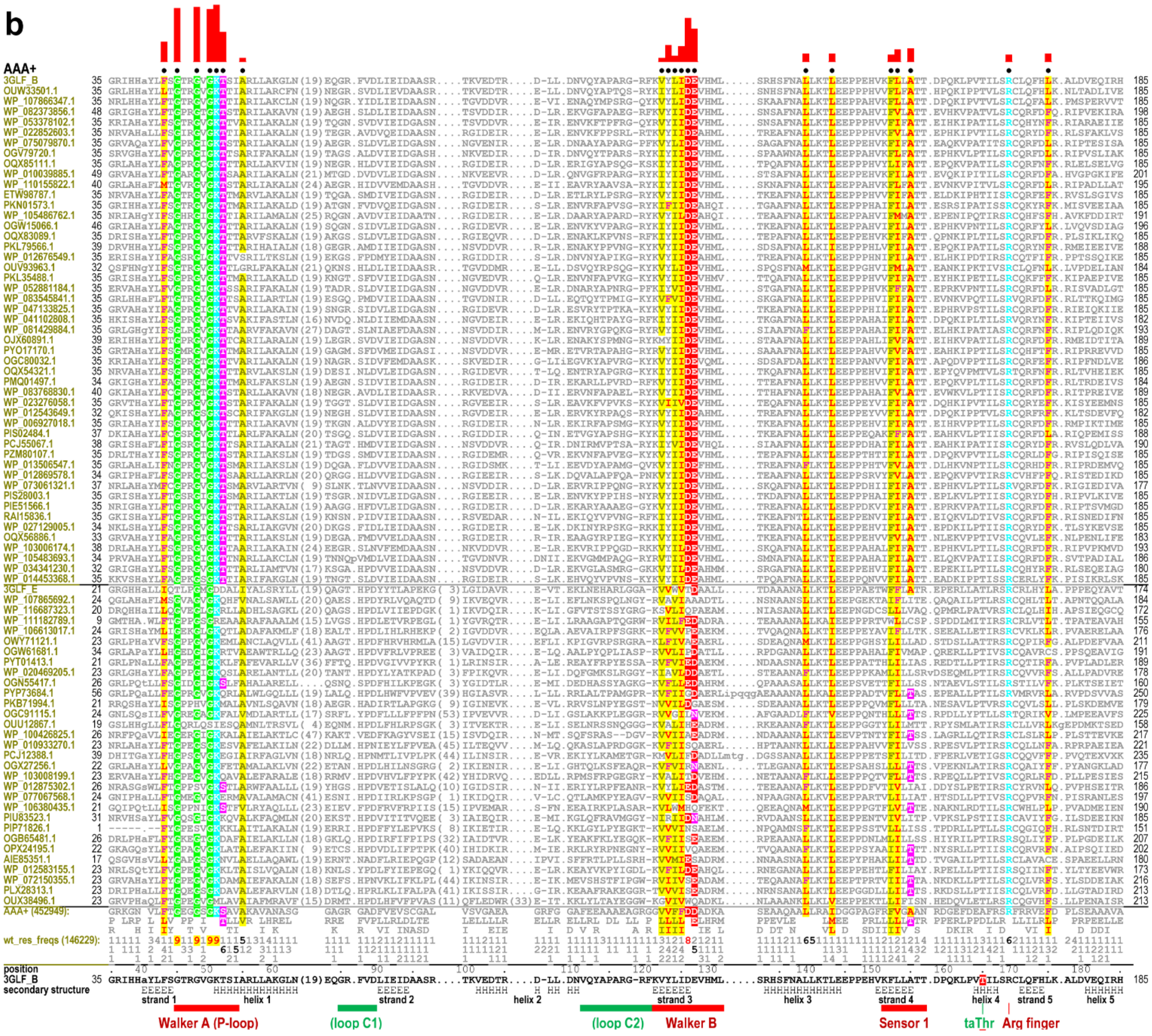

Figure S1 (cont.)

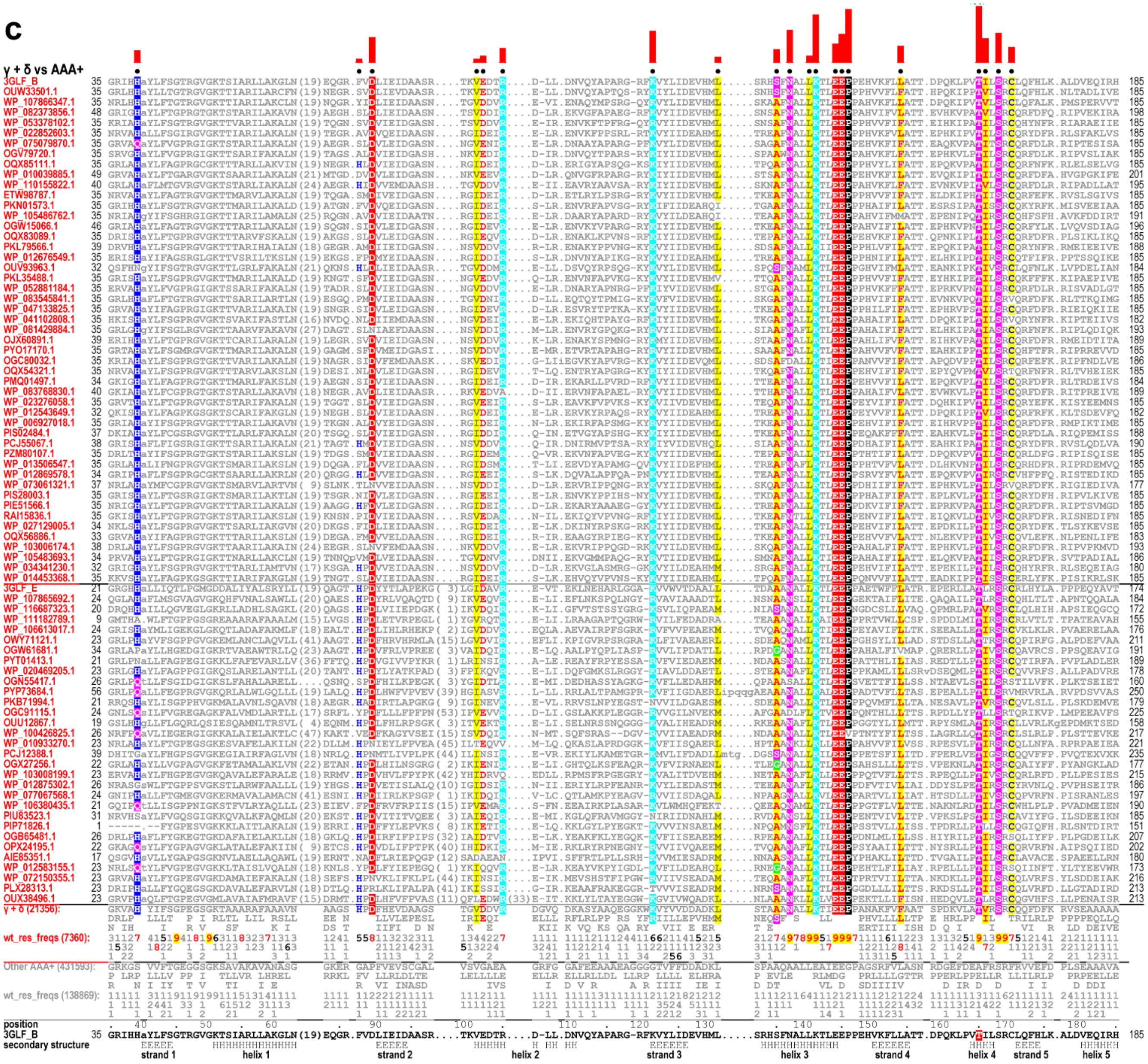

Figure S1 (cont.)

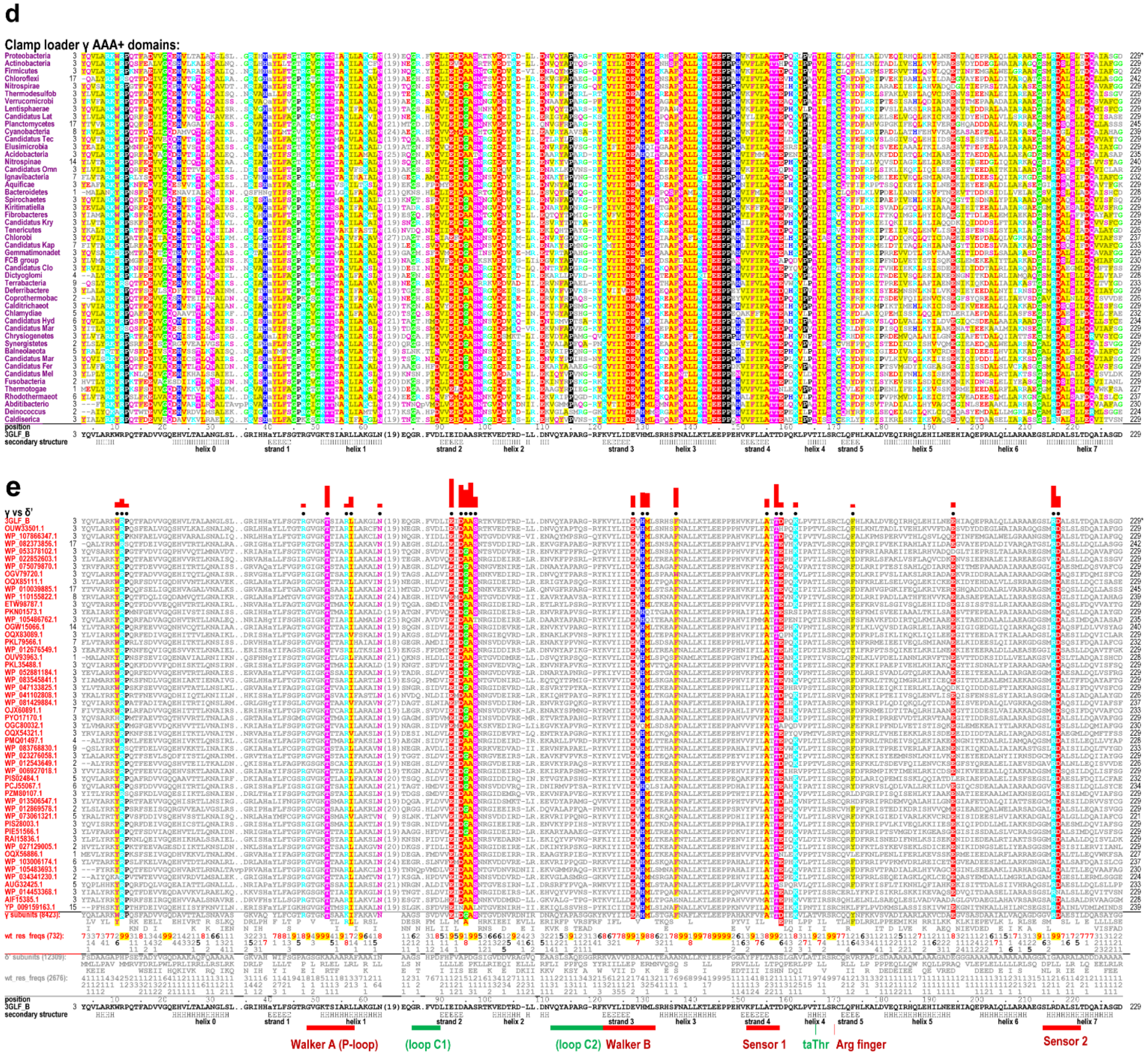

3. Figure S2.

Figure S2: DCA of AAA+ proteins fail to identify the DARC-defined clamp-loader-specific pairs given in Table 1. Shown are the top 20 DC-scoring residue pairs within Domain I (residues 42-176) based either (**a**) on a (580,241 sequence) AAA+ MSA lacking DNA clamp loader proteins or (**b**) on the (622,021 sequence) AAA+ MSA used as input to DARC. These pairs mainly occur within the structural core and fail to include the clamp-loader-specific pairs at the N-terminal ends of the α2 and α3 helices or associated with the C1 and C2 loops.

a **b**

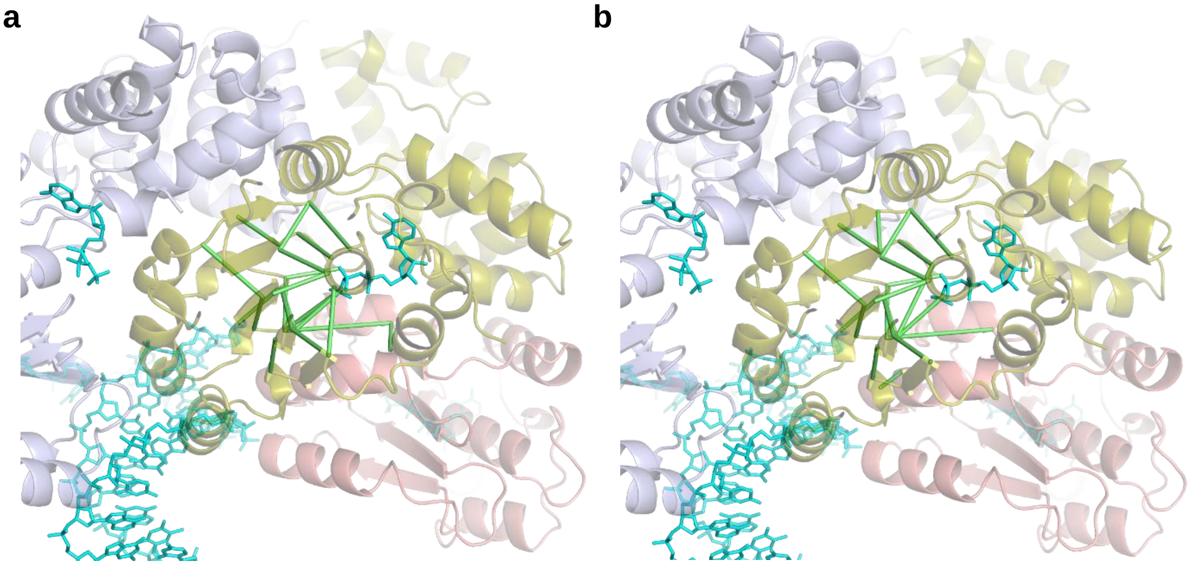

4. Figure S3.

Figure S3: PK/LDH enzyme-coupled ATP hydrolysis assay. **a.** An example of the standard curve used in ATP hydrolysis enzyme coupled assay. Known concentrations of ADP were added to the mixture for the enzyme-coupled assay except for γ-complex, DNA and ATP. The slope of the line corresponds to the fluorescent intensity/[ADP]. This slope is used to calculate the rate of ATP hydrolysis in the reaction shown in b. **b.** representative NADH emission spectra used in ATP hydrolysis assay. Emission spectra is recorded for the sample containing 1000 nM of DNA in addition to all the other reagents except for the γ-complex. The clamp loader is then added 50 s after the recording of the spectra and continued for an additional 500 s. The slope of this line is divided by the slope of the standard curve to obtain the rate of ATP hydrolysis ([ATP(uM)/s]).

a b

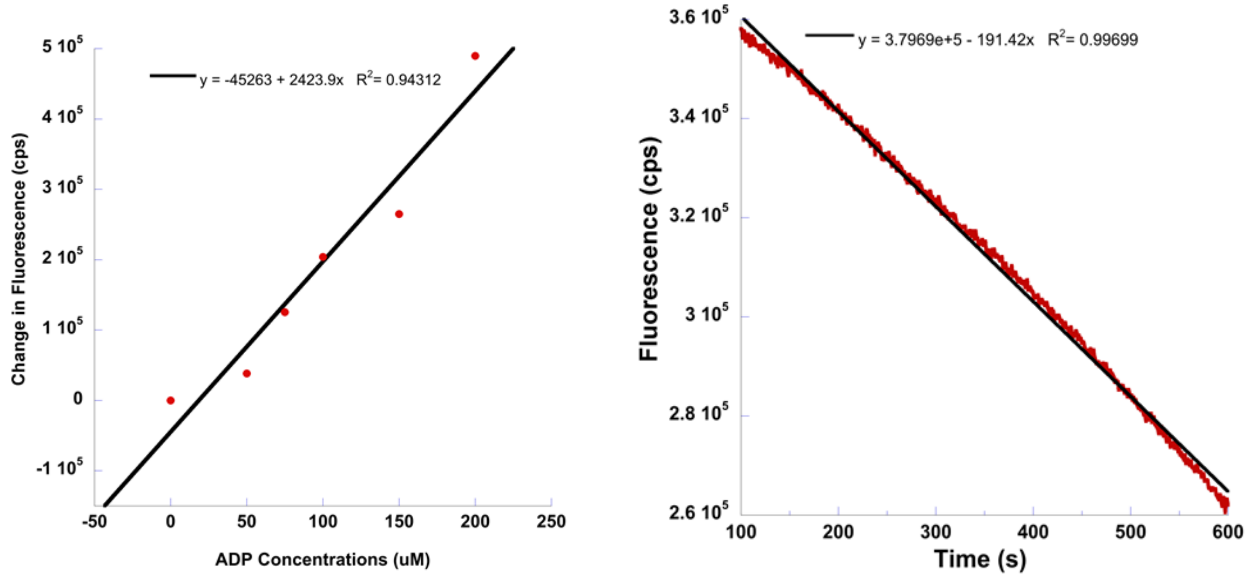

5. Table S1.

Table S1. *E. Coli* Clamp Loader structure $_{3D}S_{DC}$ and $_{3D}S_P$ scores expressed as -log$_{10}$(*p*-value).

pdb ident.	resol. (Å)	ligands	$_{3D}S_{DC}$[a]			$_{3D}S_P$			sum	ave
			γ_2 (γ/δ’)	γ_2(γ)	δ’ (γ/δ’)	γ_2 (γ/δ’)	γ_2(γ)	δ’ (γ/δ’)’	*S*	Δ*S*
3glf	3.39	Zn++, Mg++, DNA,ADP•BeF_3	262	158	241	3.5	5.7	3.2	**673**	2.7
			257	158	241	4.1	5.2	2.7	**668**	3.4
3glg	3.25	“ “ “ “ (T157A-γ mutant)	255	157	243	4.1	4.5	3.1	667	0.9
			259	157	239	4.7	4.4	2.8	667	1.8
3gli	3.5	ψ, Zn++, Mg++, DNA,ADP•BeF_3,	251	158	241	4.2	4.2	2.7	661	1.2
			254	158	235	4.1	4.7	2.7	660	0.9
1jr3	2.7	Zn++	**272**	146	223	2.9	5	3.6	652	2.9
3glh	3.89	none	**270**	141	225	2.9	5.1	3.5	648	1.4
			**268**	141	226	2.9	4.5	3.5	646	3.4
			**265**	138	223	2.9	4.6	3.6	638	2.7
1xxi	4.1	Zn++, PO_4, ADP	253	137	226	2.4	< 2	3.5	624	2.4
			249	134	225	2.4	< 2	3.5	616	-1.2
1xxh	3.45	Zn++, PO_4, ATPγS	242	142	217	3.6	4.7	< 2	612	-1.2
			244	138	214	< 2	5.4	< 2	606	0.9
		Average:	257	147	230	3.3	4.4	3	646	1.3

[a]The scores were computed using a maximum 3D distance between pairs of 3.5 Å and correspond to either the γ2 or the δ’ subunits with the subunit(s) in parentheses corresponding to the BPPS-generated subgroup alignment used to compute DCA scores.
